# The multifunctional ascorbate peroxidase MoApx1 secreted by *Magnaporthe oryzae* mediates the suppression of rice immunity

**DOI:** 10.1093/plcell/koaf146

**Published:** 2025-06-11

**Authors:** Muxing Liu, Ziqian Guo, Jiexiong Hu, Yuke Chen, Fang Chen, Weizhong Chen, Wenya Wang, Boyang Ye, Zhixiang Yang, Gang Li, Xinyu Liu, Haifeng Zhang, Ping Wang, Zhengguang Zhang

**Affiliations:** Sanya Institute of Nanjing Agricultural University, Department of Plant Pathology, College of Plant Protection, Nanjing Agricultural University, and Key Laboratory of Integrated Management of Crop Diseases and Pests, Ministry of Education, Nanjing 210095, China; Sanya Institute of Nanjing Agricultural University, Department of Plant Pathology, College of Plant Protection, Nanjing Agricultural University, and Key Laboratory of Integrated Management of Crop Diseases and Pests, Ministry of Education, Nanjing 210095, China; Sanya Institute of Nanjing Agricultural University, Department of Plant Pathology, College of Plant Protection, Nanjing Agricultural University, and Key Laboratory of Integrated Management of Crop Diseases and Pests, Ministry of Education, Nanjing 210095, China; Sanya Institute of Nanjing Agricultural University, Department of Plant Pathology, College of Plant Protection, Nanjing Agricultural University, and Key Laboratory of Integrated Management of Crop Diseases and Pests, Ministry of Education, Nanjing 210095, China; Sanya Institute of Nanjing Agricultural University, Department of Plant Pathology, College of Plant Protection, Nanjing Agricultural University, and Key Laboratory of Integrated Management of Crop Diseases and Pests, Ministry of Education, Nanjing 210095, China; Sanya Institute of Nanjing Agricultural University, Department of Plant Pathology, College of Plant Protection, Nanjing Agricultural University, and Key Laboratory of Integrated Management of Crop Diseases and Pests, Ministry of Education, Nanjing 210095, China; Sanya Institute of Nanjing Agricultural University, Department of Plant Pathology, College of Plant Protection, Nanjing Agricultural University, and Key Laboratory of Integrated Management of Crop Diseases and Pests, Ministry of Education, Nanjing 210095, China; Sanya Institute of Nanjing Agricultural University, Department of Plant Pathology, College of Plant Protection, Nanjing Agricultural University, and Key Laboratory of Integrated Management of Crop Diseases and Pests, Ministry of Education, Nanjing 210095, China; Sanya Institute of Nanjing Agricultural University, Department of Plant Pathology, College of Plant Protection, Nanjing Agricultural University, and Key Laboratory of Integrated Management of Crop Diseases and Pests, Ministry of Education, Nanjing 210095, China; Sanya Institute of Nanjing Agricultural University, Department of Plant Pathology, College of Plant Protection, Nanjing Agricultural University, and Key Laboratory of Integrated Management of Crop Diseases and Pests, Ministry of Education, Nanjing 210095, China; Sanya Institute of Nanjing Agricultural University, Department of Plant Pathology, College of Plant Protection, Nanjing Agricultural University, and Key Laboratory of Integrated Management of Crop Diseases and Pests, Ministry of Education, Nanjing 210095, China; Sanya Institute of Nanjing Agricultural University, Department of Plant Pathology, College of Plant Protection, Nanjing Agricultural University, and Key Laboratory of Integrated Management of Crop Diseases and Pests, Ministry of Education, Nanjing 210095, China; Department of Microbiology, Immunology, and Parasitology, Louisiana State University Health Sciences Center, New Orleans, LA 70112, USA; Sanya Institute of Nanjing Agricultural University, Department of Plant Pathology, College of Plant Protection, Nanjing Agricultural University, and Key Laboratory of Integrated Management of Crop Diseases and Pests, Ministry of Education, Nanjing 210095, China

## Abstract

Fungi secrete effector proteins, including extracellular redox enzymes, to inhibit host immunity. Redox enzymes have been hypothesized to inhibit host reactive oxygen species (ROS); however, how they suppress host immunity remains unknown. We characterized an extracellular ascorbate peroxidase (MoApx1) that is secreted into rice chloroplasts by the rice blast fungus *Magnaporthe oryzae*. MoApx1 displays multifunctional capabilities that significantly contribute to fungal virulence. Firstly, MoApx1 neutralizes host-derived H_2_O_2_ within the chloroplast through its peroxidase activity, thereby inhibiting chloroplast ROS (cROS)-mediated defense responses. Secondly, MoApx1 targets the photosystem I subunit OsPsaD, disrupting photosynthetic electron transport to further suppress cROS production. Most importantly, MoApx1 has evolved a fungal-specific starch-binding domain that binds host starch, inhibiting its degradation and disrupting the energy supply required for host resistance. Our findings underscore the importance of a novel multifaceted strategy, potentially widely employed by other fungal pathogens, in suppressing host immunity during host–microbe interactions.

## Teaser

The cytoplasmic effector MoApx1 suppresses immunity in rice by inhibiting chloroplast reactive oxygen species-mediated defense responses and disrupting the energy supply required for host resistance.

## Introduction

The interplay between plant pathogens and host immune systems is dynamic, and various interactive counter strategies are deployed by both sides. Among these strategies, pathogens' secretion of effector proteins, including extracellular enzymes, represents a sophisticated means to subvert plant defense mechanisms (Jones and Dangl 2006; Wang et al. 2022). The chloroplast functions as a critical hub for immune responses in plants. They are not only essential for photosynthesis but also important in the production of immune signaling molecules, including a large amount of reactive oxygen species (ROS), such as singlet oxygen (^1^O_2_), superoxide anion (O_2_·⁻), hydroxyl radical, and hydrogen peroxide (H_2_O_2_) (de Torres Zabala et al. 2015; Serrano et al. 2016; Kachroo et al. 2021). Recognizing the oxidative environment, pathogens have evolved secretory ROS-degrading enzymes to scavenge H_2_O_2_. However, none of the ROS-defective mutant strains showed altered pathogenicity (Schouten et al. 2002; Robbertse et al. 2003; Tanabe et al. 2011), suggesting that merely scavenging ROS may be insufficient to suppress host immunity and additional enzymes or functions are needed. In plants, H_2_O_2_ can be scavenged by several antioxidant enzymes, including catalases, ascorbate peroxidases (APXs), peroxiredoxins, and glutathione S-transferases (Tanabe et al. 2011). Among them, APXs prove critical in catalyzing the reduction of H_2_O_2_ to water via the electron donor ascorbate (Maruta et al. 2016); however, how APXs secreted by pathogenic fungi scavenge host H_2_O_2_ remain not characterized.

ROS generated in chloroplasts (cROS) is an important contributor to host immunity. The photosynthetic electron transport chains of photosystems I and II (PSI and II) in the chloroplast are significant players in providing electrons for free radical formation. The most prominent cROS generation routes are oxygen photoreduction at PSI and possibly via the PSII electron acceptor plastoquinone (Vetoshkina et al. 2017). PSII mainly forms singlet oxygen ^1^O_2_, while PSI produces superoxide anion O_2_·⁻ that is converted to H_2_O_2_ (Kangasjarvi et al. 2014; Trotta et al. 2014). Photosystems reinforce the importance of the chloroplast in integrating photosynthesis and defense signals. For example, during the rice blast fungus *Magnaporthe oryzae*–rice interaction, the phosphorylated LHCB5, a light-harvesting complex protein in PSII, forms a trimeric complex that disrupts its binding with the PSII subunit PsbS, resulting in decreased electron transfer, increased cROS, and enhanced basal resistance (Liu et al. 2019). For successful colonization, plant pathogens secrete effectors that target the chloroplast, thereby hindering the defense ability of the host (de Torres Zabala et al. 2015; Kretschmer et al. 2019; Littlejohn et al. 2021). For example, HopN1, secreted by the bacterium *Pseudonomas syringae*, degrades the PSII subunit PsbQ, reducing photosynthesis and attenuating cROS production (Lopez-Solanilla et al. 2004; Rodriguez-Herva et al. 2012). However, whether such effectors specifically target PSI to inhibit immunity remains uncharacterized.

Chloroplasts are the primary sites for starch synthesis. During photosynthesis, plants convert light energy into chemical energy through the synthesis of organic compounds, such as glucose, which can then be converted into starch for storage in the endosperm (Scofield et al. 2009; Bahaji et al. 2014). Starch can also be found in leaves. During the day, excessive photosynthesis results in the accumulation of starch in chloroplasts, which can then be degraded at night to sustain plant growth (Dong et al. 2024; Yan et al. 2024). However, whether leaf starch plays a role in regulating disease resistance and, likewise, if pathogens interfere with starch-regulated resistance remains unknown.

Here, we identified an extracellular ascorbate peroxidase, MoApx1, from *M. oryzae*. We found that MoApx1 eliminates leaf H_2_O_2_ through its APX activity. In addition, MoApx1 functions as an effector by targeting the PSI subunit PsaD to inhibit electron transport that suppresses host immunity. Interestingly, the C-terminal starch-binding domain (SBD) of MoApx1 enables it to bind to starch and inhibit its degradation, potentially further disrupting energy supply and suppressing host immunity.

## Results

### *M. oryzae* secretes ascorbate peroxidase MoApx1 to promote virulence

In previous studies to examine the mechanisms of effector transport in *M. oryzae*, we found that MoSwa2 functions as a disassembly factor to mediate COPII trafficking and participates in ROS scavenging by regulating the secretion of extracellular enzymes (Liu et al. 2021). To further explore the molecular mechanism by which MoSwa2-regulated secreted enzymes inhibit host immunity, we conducted indiscriminate single knockouts of each of these extracellular enzyme-encoding genes (Liu et al. 2021 ). We identified MGG_09398 that encodes an APX homolog named MoApx1. Using RT-qPCR to examine the expression pattern of *MoAPX1* in susceptible rice cultivar ‘CO39’ inoculated with wild-type *M. oryzae* strain Guy11 at different infectious stages, we found that *MoAPX1* is highly expressed at 24 and 48 h post-infection (hpi) (Supplementary Fig. S1A), suggesting a potential role in fungal virulence. We then obtained a Δ*Moapx1* mutant strain and characterized its phenotype following verification by Southern blot analysis (Supplementary Fig. S2). There were no signiﬁcant differences between the Δ*Moapx1* mutant and Guy11 or the complemented Δ*Moapx1*/*MoAPX1* strain in growth, conidiation, conidial germination, or appressoria formation (Supplementary Table S1).

**Figure 1.**
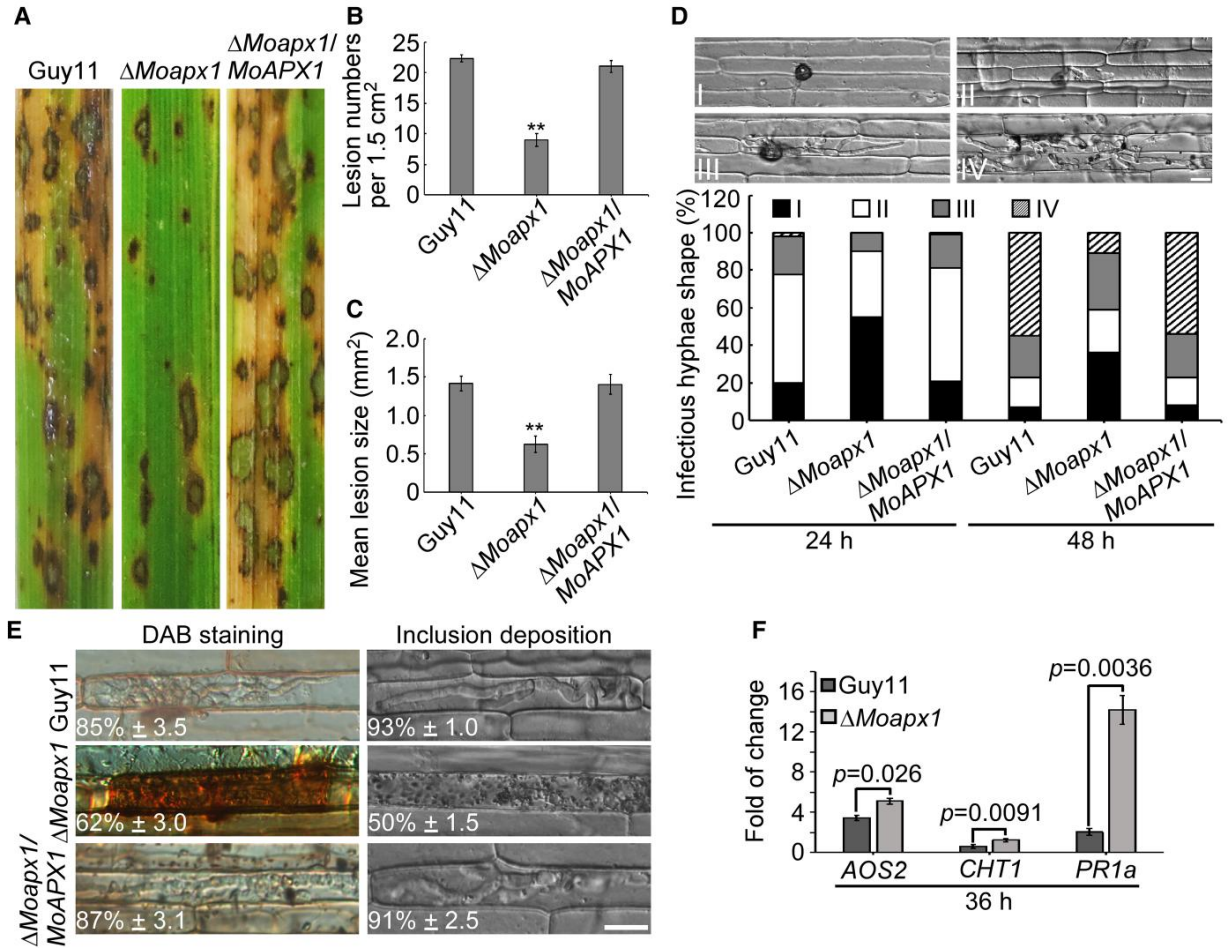
MoApx1 is required for the virulence of the rice blast fungus. **A)** MoApx1 is required for the full virulence of *M. oryzae*. The virulence was tested using the conidial suspension spray assay. Diseased rice leaves were photographed after 7 d post-inoculation (dpi). Scale bar, 10 *µ*m. **B and C)** The disease lesion numbers and the lesion sizes in **A)** were measured. The mean values of 3 determinations with Sds are shown. Significant differences were determined by Student's *t*-test and marked with asterisk (*P* < 0.01). **D)** MoApx1 is important for normal invasion growth. Invasive hyphae were examined at 24 and 48 hpi. The experiments were repeated independently with similar results at least 3 times. Scale bar, 10 *µ*m. **E)** The Δ*Moapx1* mutant cannot inhibit ROS bursts in rice cells. DAB (3,3′-diaminobenzidine) staining and inclusion observation on infected leaf sheaths at 24 and 48 hpi. The experiments were repeated independently with similar results at least 3 times. Scale bar, 10 *µ*m. **F)** The Δ*Moapx1* mutant infection induced the PR gene expression in rice. Examination of transcript levels of *AOS2*, *CHT1*, and *PR1a* genes in rice cultivar ‘CO39’ inoculated with the Guy11 and the Δ*Moapx1* mutant at 36 h. The error bars represent Sd (*n* = 3). Significant differences were determined by 2-sided Duncan's new multiple-range tests, and marked with *P* values (*P* < 0.05).

To further examine if MoApx1 is required for virulence, conidial suspensions from Guy11, Δ*Moapx1*, and the complemented strains were sprayed onto susceptible rice cultivar ‘CO39’ seedlings. We found that the Δ*Moapx1* mutant is severely attenuated in virulence, with the reduction of leaf lesion numbers and lesion sizes by >60% compared with controls (Fig. 1, A to C). Further rice sheath infection assays showed that invasive hyphae (IH) growth was significantly restricted at 24 hpi in the Δ*Moapx1* mutant (>85%, *n* = 100), and the IH failed to expand to adjoining cells even at 48 hpi (>80%, *n* = 100) (Fig. 1D). The above results indicated that MoApx1 mainly functions in IH growth and lesion formation.

To examine whether MoApx1 inhibits the host immune response associated with the burst of host H_2_O_2_, we used 3,3′-diaminobenzidine (DAB) staining to estimate H_2_O_2_ production in infected rice sheathes. H_2_O_2_ was rarely found in infection by Guy11 and the complemented strain but readily detected in cells infected with the Δ*Moapx1* mutant (Fig. 1E). Strikingly, most of the Δ*Moapx1* mutant infected sites (>50%, *n* = 100) exhibited a strong defense response. There were lots of dark and brown inclusions attached to the IH of the Δ*Moapx1* mutant at 32 hpi, compared to those by Guy11 (<10%, *n* = 100) (Fig. 1E). Moreover, the expression of pathogenesis-related (PR) genes *AOS1* (allene oxide synthase), *CHT1* (chitinase), and *PR1a* (pathogenesis-related gene) was highly induced at 36 hpi after Δ*Moapx1* infection, compared to that by Guy11 (Fig. 1F).

To determine whether MoApx1 directly eliminates H_2_O_2_, we expressed MoApx1 in vitro and found that it exhibits peroxidase activities (Supplementary Fig. S3, A and B). Importantly, we found that there are 7 types of nonsynonymous sequence mutations and 1 deletion mutant in MoApx1 coding sequences from different host-speciﬁc isolates, including *Oryza sativa*, *Eleusine indica*, *Triticum aestivum*, *Setaria viridis*, and *Lolium* (Zhong et al. 2016) (Supplementary Fig. S3A and Data Set S1). Further tests showed 2 mutations (A121V and L246P) with reduced enzymatic activities compared to MoApx1 (Supplementary Fig. S3B). In addition, 2 enzymatic activity sites are critical for virulence and IH growth (Supplementary Fig. S3, C to E). These data indicated that MoApx1 is essential for the virulence of the rice blast fungus.

### MoApx1 is secreted into rice chloroplast

**Figure 2.**
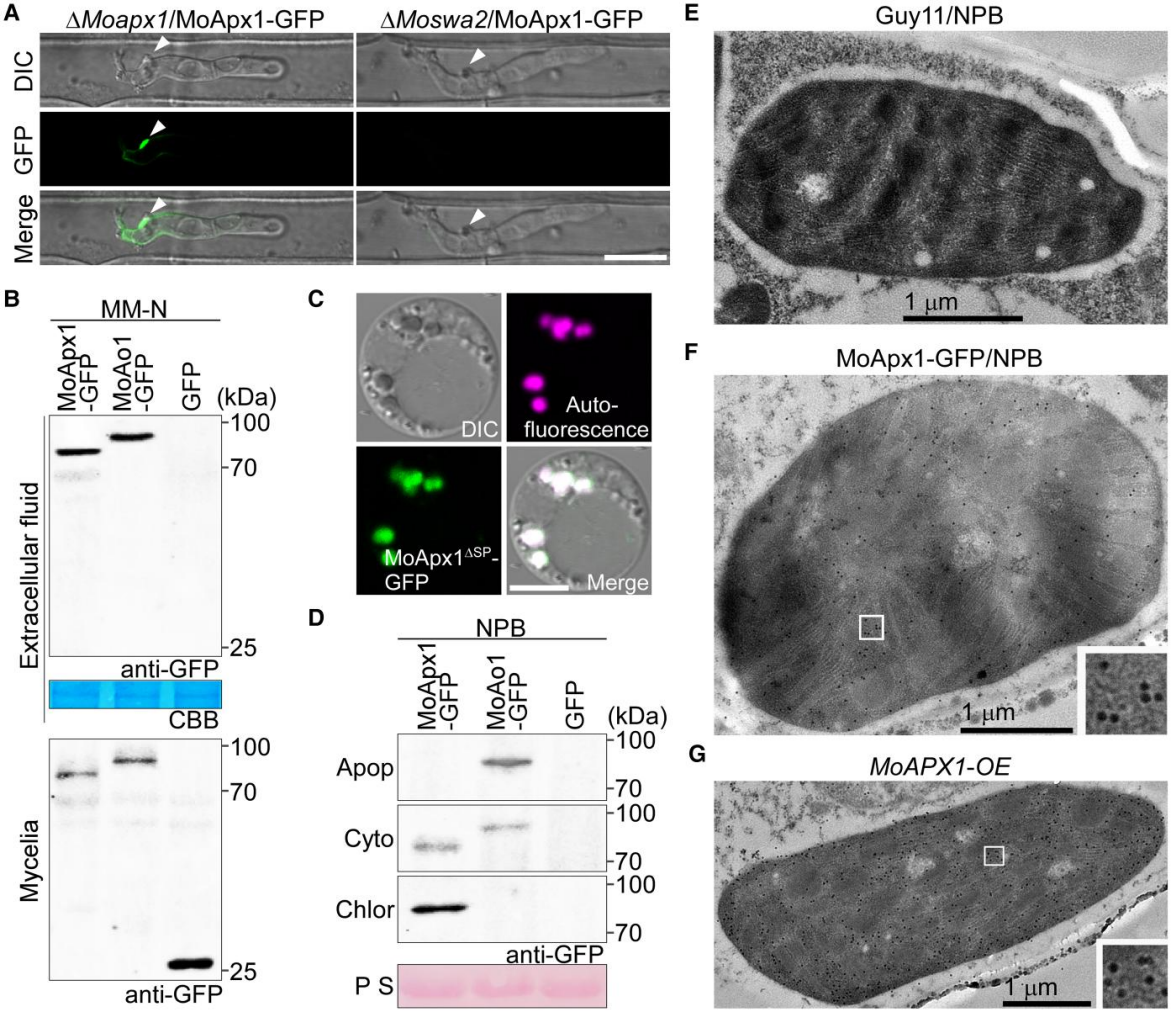
MoApx1 is a cytoplasmic effector secreted into the rice chloroplast. **A)** MoApx1 is a cytoplasmic effector secretory regulated by MoSwa2. The fungal transformants Δ*Moapx1* and Δ*Moswa2* expressing MoApx1:GFP at 30 hpi in the sheath cells of rice cultivar ‘CO39’ rice are shown as a projection of a confocal microscope. Arrows indicate the biotrophic interface complex. Scale bar, 10 *µ*m. **B)** MoApx1 is secreted into extracellular space. The GFP-tagged MoApx1, MoAo1, and empty-GFP were expressed in the Guy11 strain, respectively. Total extracellular proteins were extracted from cultures respectively grown in MMN liquid culture for 4 d, and separated by SDS-PAGE followed by detection with the anti-GFP antibody. Coomassie brilliant blue (CBB) was used as the loading control. **C)** MoApx1 localizes in the chloroplast. GFP-tagged MoApx1 without signal peptide was expressed in the rice protoplast. The autofluorescence represents chloroplast. Scale bar, 5 *µ*m. **D)** MoApx1 targets rice chloroplast. After the susceptible rice variety NPB inoculated with above strains for 48 h, the extracellular fluids, cytoplasmic, and chloroplast proteins were isolated. The proteins were extracted and separated by SDS-PAGE followed by detection with the anti-GFP antibody. Ponceau S (PS) was used as the loading control. **E to G)** MoApx1 can enter into rice chloroplast during infection stage. The wild-type strain Guy11 and Guy11/MoApx1-GFP transformed strain were inoculated to wild-type rice NPB or *MoAPX1-OE* rice leaves at 48 hpi, the immunogold labeling results showed that MoApx1-GFP gold particles were localized in rice chloroplasts. Scale bars, 1 *μ*m.

There are 2 distinct secretion systems in *M. oryzae*. Cytoplasmic effectors are preferentially accumulated in the biotrophic interfacial complex (BIC) once delivered into the host (Khang et al. 2010; Giraldo et al. 2013). To examine the secretion of MoApx1 in rice cells, a native *MoAPX1* promotor-driven *MoAPX1*-*GFP* fusion gene was introduced into the Δ*Moapx1* and Δ*Moswa2* mutants, respectively. Based on the observation of approximately 100 infection sites, over 80% showed fluorescence accumulation in the BIC of the Δ*Moapx1* mutant, whereas this accumulation was not observed in the Δ*Moswa2* mutant (Fig. 2A). This indicated that MoApx1 is a cytoplasmic effector whose secretion is likely regulated by MoSwa2. To confirm MoApx1 is secreted into the extracellular space, the native promotor-driven *MoAPX1*-*GFP*, the control apoplastic effector *MoAO1*-*GFP* (Hu et al. 2022), and the control empty-GFP fusion genes were generated and transformed into Guy11, respectively. Secreted proteins were collected from cultures grown in liquid nitrogen starvation minimal medium (MMN), which induces protein secretion similar to the early infection process (Talbot et al. 1993, 1997; Wang et al. 2011). MoApx1 and MoAo1 were detected in cultures of MMN, but not empty-GFP (Fig. 2B), suggesting that MoApx1 is secreted into the extracellular space.

To further determine the localization of MoApx1 in the host, the GFP-tagged MoApx1 without a signal peptide (MoApx1^ΔSP^-GFP) was transiently expressed in rice protoplasts. The co-localization of GFP fluorescence with autofluorescence of chloroplasts confirmed that MoApx1 is localized within the chloroplast (Fig. 2C). To test whether MoApx1 is secreted into the chloroplast during *M. oryzae* infection, we isolated the apoplast, cytoplasm, and chloroplast proteins of the ‘Nipponbare’ (NPB) background variety inoculated with *M. oryzae* strains expressing MoApx1-GFP, MoAo1-GFP, and empty-GFP at 48 hpi, respectively. We found that MoApx1 mainly accumulates in the chloroplast but not the apoplast or the cytoplasm (Fig. 2D). Using immunogold electron microscopy, we examined the localization of MoApx1-GFP in rice at 48 hpi and observed a substantial accumulation of gold particles in chloroplasts, but not in Guy11 infected rice leaves (Fig. 2, E and F). This result was further validated using transgenic rice lines that overexpress MoApx1 without a signal peptide (*MoAPX1*-*OE*) (Fig. 2G and Supplementary Fig. S4A). These findings indicated that MoApx1 is capable of being secreted into rice chloroplasts, where it suppresses the plant's immune response.

### MoApx1 suppresses host immunity by competitively binding to the PSI subunit PsaD

**Figure 3.**
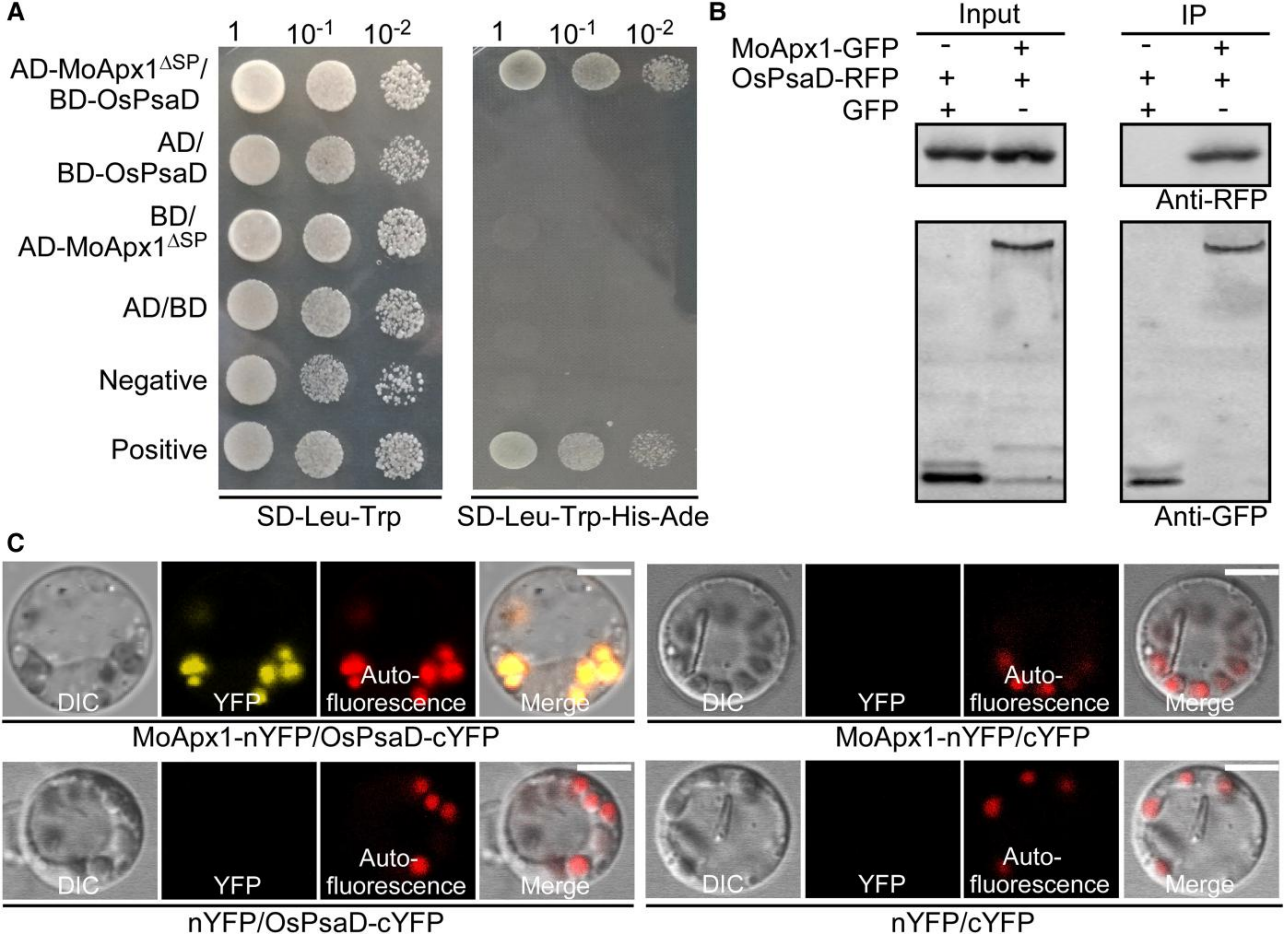
Interactions between MoApx1 and OsPsaD in vitro and in vivo. **A)** Yeast 2-hybrid assay between AD (pGADT7)-MoAxp1^ΔSP^ (without the signal peptide sequence) and BD (pGBKT7)-OsPsaD. Cells were plated on a SD-Leu-Trp medium and then transferred onto SD-Ade-Leu-Trp-His medium. **B)** Co-IP analysis of *MoAPX1*^ΔSP^-*GFP* and *OsPsaD*-*RFP* in vivo. The *MoAPX1*-*GFP* or empty-*GFP* was co-expressed with *OsPSAD*-*RFP* genes in rice protoplast. The co-IP experiment was performed with the anti-GFP antibody, and the isolated protein was analyzed by immunoblot using an anti-RFP antibody to detect OsPsaD and an anti-GFP antibody to detect MoApx1. The experiments were repeated independently with similar results at least 3 times. **C)** Bimolecular Fluorescence Complementation (BiFC) assays in rice protoplast cells. Co-expression of MoApx1^ΔSP^-nYFP and OsPsaD-cYFP showed that MoApx1 and OsPsaD co-localized in chloroplast. The relevant negative controls showed no fluorescence. Autofluorescence represents chloroplast. All the experiments were repeated independently with similar results at least 3 times. Scale bar, 5 *µ*m.

To determine whether MoApx1 functions as an effector that targets and inhibits key components of host immunity, we constructed a yeast 2-hybrid (Y2H) bait vector for MoApx1 (pGBKT7-*MoAPX1*^ΔSP^) and screened a rice cDNA library. We identified many chloroplast proteins, with LOC_Os08g44680 (OsPsaD, a PSI subunit) appearing most frequently (Supplementary Table S2). We then verified the MoApx1–OsPsaD interaction by a Y2H repeat and co-immunoprecipitation (co-IP) using proteins extracted from rice protoplasts (Fig. 3, A and B). To further examine the location of MoApx1–OsPsaD interaction, we generated an N-terminal YFP (MoApx1-nYFP) and a C-terminal YFP (OsPsaD-cYFP) fusion proteins and co-expressed them in rice protoplasts. The result confirmed that the interaction mainly occurs in the chloroplast (Fig. 3C), consistent with the localization results of MoApx1 (Fig. 2).

**Figure 4.**
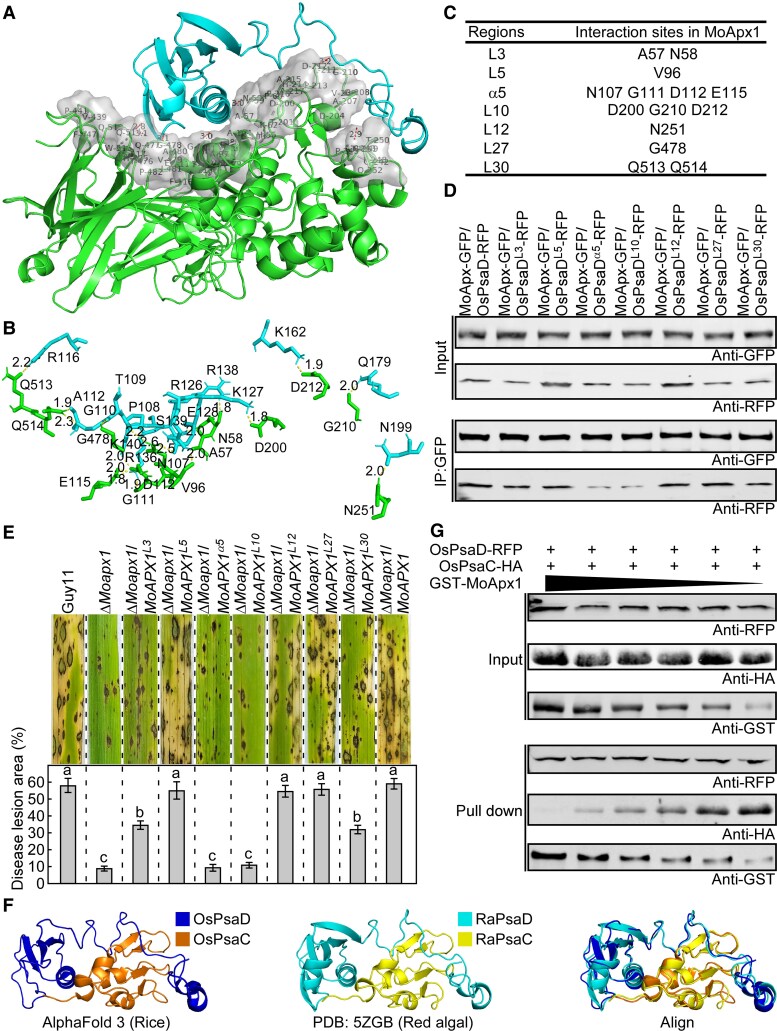
MoApx1 suppresses host immunity by competitively binding OsPsaD. **A to C)** A structural model for the MoApx1–OsPsaD interaction, predicted by ClusPro 2.0 server. The surface of the MoApx1–OsPsaD and the interaction amino residues were visualized by PyMOL. The yellow dotted line with the marked distance is hydrogen binding. L, loop; α, α-helix. **D)** α5 and L10 regions are required for the MoApx1–OsPsaD interaction. In vivo co-IP assay between OsPsaD with MoApx1 or interaction sites mutants. The MoApx1-GFP or mutants were co-expressed with OsPsaD-RFP genes in rice protoplast. The co-IP experiment was performed with the anti-GFP antibody, and the isolated protein was analyzed by immunoblot using an anti-RFP antibody to detect OsPsaD and an anti-GFP antibody to detect MoApx1. **E)** The α5 and L10 regions in MoApx1 are required for the full virulence of *M. oryzae*. The virulence was tested using the conidial suspension spray assay. Diseased rice leaves were photographed after 7 dpi. The disease lesion area was assessed using ImageJ software. The mean values of 3 determinations with Sds are shown. Significant differences were determined by 2-sided Duncan's new multiple-range tests, and marked with different letters (*P* < 0.01). **F)** The structural model of OsPsaC–OsPsaD. The OsPsaC–OsPsaD model generated using AlphaFold 3, closely resembles the structure found in red algae (PDB ID: 5ZGB). The cartoon structures were visualized by PyMOL. **G)** In vitro competitive binding assay between MoApx1 and OsPsaC with OsPsaD. The RFP-tagged OsPsaD and HA-tagged OsPsaC were expressed and purified from rice protoplasts. GST-MoApx1 was purified from *E. coli BL21* (DE3) cell lysate. Equal amounts of OsPsaD-RFP and OsPsaC-HA were added to each reaction, along with a gradient dilution of GST-MoAxp1, and the mixtures were enriched using RFP beads. Eluted proteins were analyzed by immunoblot with anti-RFP, anti-HA, and anti-GST antibodies.

Moreover, we carried out molecular modeling and docking analysis to further identify the amino acids of MoApx1 required for binding to OsPsaD. 3D models of MoApx1 and OsPsaD were obtained from AlphaFold 3 calculations, and most regions were confident with pLDDT > 90 and pTM > 0.6 (Supplementary Fig. S5). ClusPro was used to predict that interactions between MoApx1 loop3 (L3), loop5 (L5), α-helix 5 (α5), loop10 (L10), loop12 (L12), loop27 (L27), and loop30 (L30) with OsPsaD (Fig. 4A). We also predicted that the amino acid residues in these loops or helix that form hydrogen bonds (yellow dotted line) with correspondent residues in OsPsaD (Fig. 4, B and C).

To verify this modeling prediction, alanine substitution in each of the 7 regions was carried out. Co-IP revealed that mutations in α5 (positions 107, 111, 112, and 115) and L10 (positions 200, 210, and 212) nearly abolish the MoApx1–OsPsaD interaction. In addition, L3 (positions 57 and 58) and L30 (positions 513 and 514) are also required for full binding activities (Fig. 4D). Moreover, natural mutations in MoApx1 do not affect the MoApx1–OsPsaD interaction (Supplementary Fig. S6). Notably, mutations in α5 and L10 resulted in a sharp decrease in virulence, similar to Δ*Moapx1* (Fig. 4E). In contrast, mutations in L3 and L30 caused a moderate reduction in virulence, while mutations at other sites maintained normal pathogenicity, comparable to control strains (Fig. 4E).

PsaD contributes to the binding of the PsaC subunit to the PSI core complex and plays a significant role in the efficient transfer of electrons from PsaC to ferredoxin (Klukas et al. 1999; Fromme et al. 2001; Pi et al. 2018). Strikingly, we noticed that the region where OsPsaD binds to MoApx1 coincides with its binding site for OsPsaC, leading us to hypothesize that this will prevent OsPsaC from properly binding to OsPsaD (Fig. 4F). To test this hypothesis, we expressed and purified RFP-tagged OsPsaD and HA-tagged OsPsaC proteins in rice protoplasts and the GST-tagged MoApx1 protein in *Escherichia coli*. An in vitro competitive binding assay showed that MoApx1 reduces the binding affinity between OsPsaD and OsPsaC in a dose-dependent manner (Fig. 4G). Moreover, MoApx1 did not interact with OsPsaC (Supplementary Fig. S7). These results suggested that MoApx1 competes with OsPsaC in binding to OsPsaD.

### OsPsaD positively regulates rice immunity against *M. oryzae* infection

To examine whether OsPsaD regulates resistance against the blast pathogen, we determined *OsPsaD* expression during various growth stages by quantitative RT-PCR. *OsPsaD* was highly expressed at 48 hpi following infection by Guy11 (Supplementary Fig. S1B), indicating its potential role in rice resistance. We then generated the overexpression (*OsPsaD*-*OE*) and CRISPR-Cas9-edited (*Ospsad*-*KO*) transgenic rice lines in the NPB background. These overexpression lines were characterized by Western blot analysis, and the CRISPR-Cas9-edited lines were characterized by PCR and DNA sequencing (Supplementary Fig. S4, B to D). None of the transgenic lines showed visible differences in plant growth and yield (Supplementary Fig. S4E).

**Figure 5.**
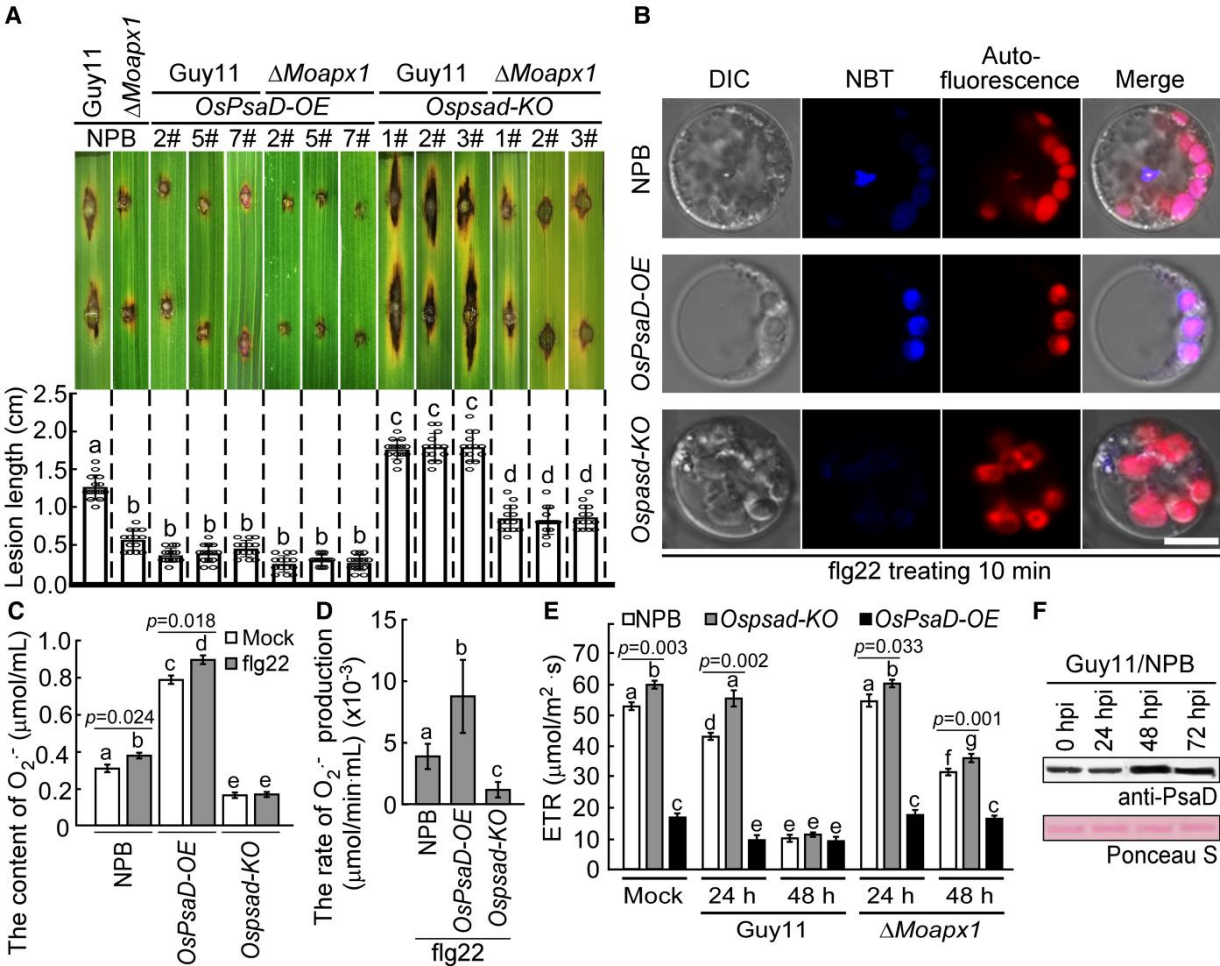
Determination of resistance of OsPsaD knockout and overexpression transgenic lines. **A)** Infection phenotype of *Ospsad-KO* and *OsPsaD-OE* lines against *M. oryzae.* The leaves of 3-wk-old plants were infected with the wild-type Guy11 and the Δ*Moapx1* strains using punch inoculation. Photos were taken at 7 dpi. The lesion length was measured by rule. The mean values of 3 measurements with Sds are shown. Significant differences were determined by 2-sided Duncan's new multiple-range tests, which were marked with different letters (*P* < 0.05). **B)** The OsPsaD overexpression lines accumulate a higher ion of superoxide anions when treated with an elicitor. The NPB, *Ospsad-KO*, and *OsPsaD-OE* rice plants treated with 0.1 *μ*m flg22 were stained with NBT. The red autofluorescence represents chloroplast. Scale bar, 5 *µ*m. **C and D)** The determination of O2.⁻. The content and production rate in rice plants when treated with or without flg22 were determined using the Superoxide Anion Content Assay Kit. The mean values of 3 measurements with Sds are shown. Significant differences were determined by 2-sided Duncan's new multiple-range tests and marked with different letters (*P* < 0.05). **E)** The determination of ETR. The ETR in NPB, *Ospsad-KO*, and *OsPsaD*-*OE* rice plants infected by Guy11 and Δ*Moapx1* at 24 and 48 hpi was measured using the instrument DUAL-PAM-100. The mean values of 3 measurements with Sds are shown. Significant differences were determined by 2-sided Duncan's new multiple-range tests and marked with different letters (*P* < 0.05). **F)** The translation of OsPsaD was induced by the infection of *M. oryzae*. The protein level of OsPsaD in the different infectious stages was determined by immunoblotting using anti-PsaD polyclonal antibodies (AS09461, Agrisera). Protein loading is indicated with Ponceau staining.

We then tested the resistance of transgenic lines to Guy11 and the Δ*Moapx1* mutant by punch inoculation. Compared with wild-type NPB, the *OsPsaD*-*OE* (2#, 5#, and 7#) rice lines showed enhanced resistance, producing smaller lesions. Notably, lines with targeted gene deletions of *Ospsad*-*KO* (1#, 2#, and 3#) were more susceptible to Guy11 and partially susceptible to the Δ*Moapx1* mutant (Fig. 5A). This suggested that *M. oryzae* secretes MoApx1 to inhibit OsPsaD-mediated rice resistance. PsaD is a component of PSI, which is primarily responsible for the production of superoxide anions (Klukas et al. 1999; Fromme et al. 2001; Kangasjarvi et al. 2014; Pi et al. 2018). To examine the resistance mechanism regulated by OsPsaD, we used the NBT staining method to assess the accumulation of O_2_·⁻ in the chloroplasts of transgenic materials treated with the elicitor flg22. The results showed that *OsPsaD*-*OE* rice lines led to higher levels of O_2_·⁻ (Fig. 5B).

To validate the accumulation of O_2_·⁻ in the chloroplasts, we measured photosynthetic parameters in the NPB, *OsPsaD*-*OE*, and *Ospsad*-*KO* rice lines treated with or without flg22 using a Chlorophyll Fluorescence Imager (Ecotek, Beijing, China). Treatment with flg22 significantly increased O_2_·⁻ production in both NPB and *OsPsaD*-*OE* rice lines compared to untreated controls, while no significant difference was observed in the *Ospsad*-*KO* rice lines. Furthermore, regardless of treatment, *OsPsaD*-*OE* rice lines exhibited a markedly higher accumulation of O_2_·⁻ than both NPB and *Ospsad*-*KO* rice lines. The content and production rate of O_2_·⁻ were significantly higher in *OsPsaD*-*OE* lines but lower in *Ospsad*-*KO* lines compared to NPB (Fig. 5, C and D). To further verify that OsPsaD is not involved in the induction of ^1^O_2_, we performed ^1^O_2_ tracking in protoplast using the SOSG probe, with Rose Bengal as a positive control (Gutierrez et al. 2014). The results demonstrated that, similar to NPB, neither *OsPsaD*-*OE* nor *Ospsad*-*KO* rice lines induced ^1^O_2_ accumulation under flg22 treatment (Supplementary Fig. S8). Additionally, the electron transport rate (ETR) was significantly enhanced in *OsPsaD*-*OE* lines and reduced in *Ospsad*-*KO* lines compared to NPB when inoculated with *M. oryzae* at 24 and 48 hpi (Fig. 5E).

We also tested the susceptibility of *MoAPX1*-*OE* rice lines to Guy11 and the Δ*Moapx1* mutant by punch inoculation. Compared with NPB, the *MoAPX1*-*OE* rice lines were more susceptible to Guy11, and the Δ*Moapx1* mutant was able to restore the virulence defect on the *MoAPX1*-*OE* rice lines (Supplementary Fig. S9, A and B). We further found the O_2_·⁻ content is not induced in *MoAPX1*-*OE* lines compared to NPB when treated with flg22 (Supplementary Fig. S9, C and D). The production rate of O_2_·⁻ in *MoAPX1*-*OE* lines was significantly slower than in NPB when treated with flg22 (Supplementary Fig. S9E). ETR was markedly reduced in *MoAPX1*-*OE* lines compared to NPB when inoculated with Guy11 or the Δ*Moapx1* mutant at 24 and 48 hpi (Supplementary Fig. S9F). We analyzed OsPsaD protein levels during *M. oryzae* infection using anti-PsaD polyclonal antibodies and observed a significant in OsPsaD protein levels 48 hpi (Fig. 5F), consistent with its transcriptional response during infection, indicating that OsPsaD is involved in the response to *M. oryzae* infection. Additionally, we analyzed OsPsaD protein levels in *MoAPX1*-*OE* lines under both infected and uninfected conditions. We found no reduction compared to the control (Supplementary Fig. S9G), suggesting that MoApx1 does not affect OsPsaD protein levels. Taken together, these findings indicated that MoApx1 targets OsPsaD to inhibit PSI-mediated cROS generation and immunity.

### MoApx1 binds starch and inhibits starch-mediated resistance against *M. oryzae* infection

**Figure 6.**
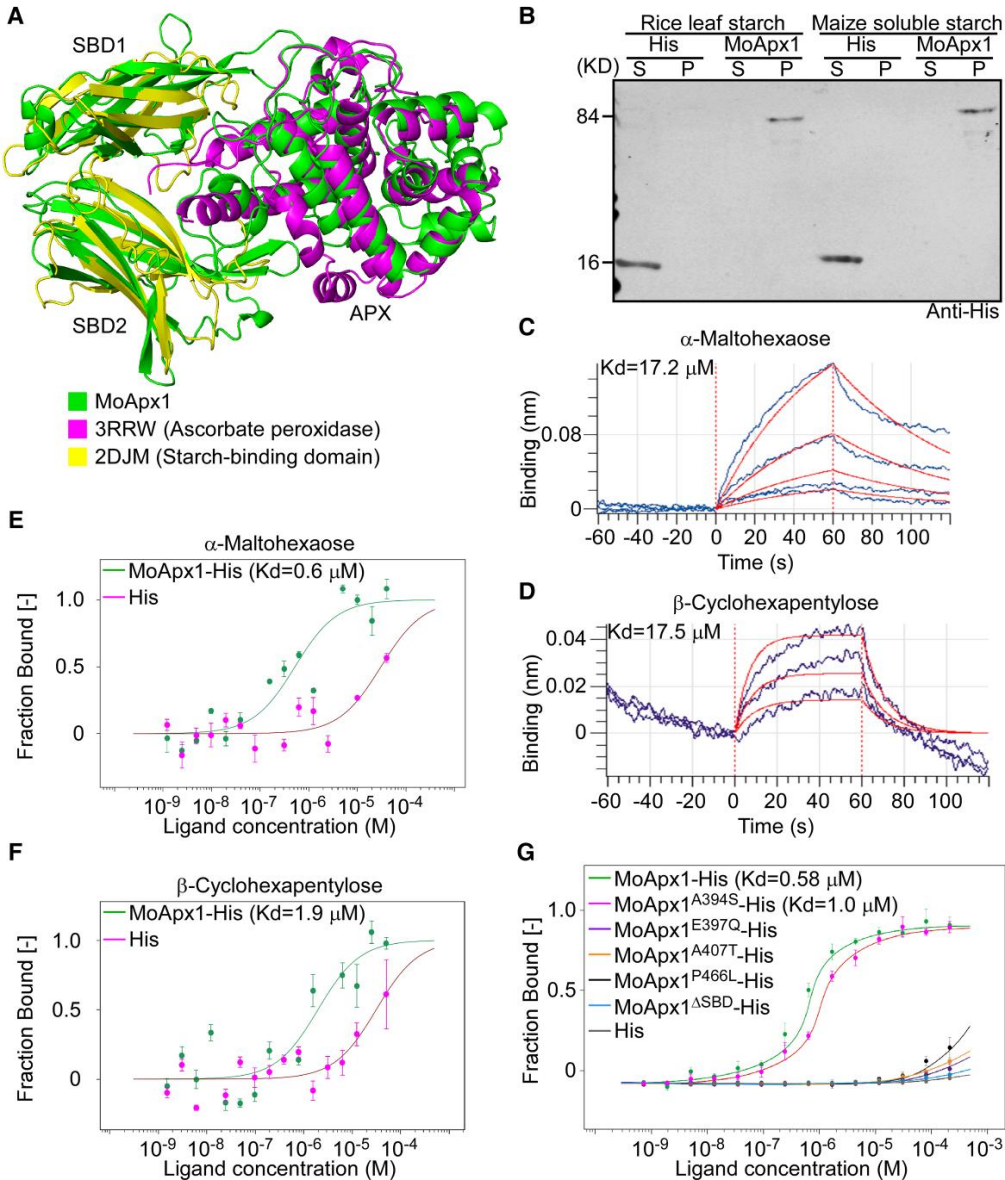
MoApx1 contains a C-terminal starch-binding domain and binds to starch. **A)** The structure model of MoApx1 was predicted by AlphaFold2, and aligned with the ascorbate peroxidase (PDB ID: 3RRW) from *Arabidopsis thaliana* and the starch-binding domain of glucoamylase from *Rhizopus oryzae* (PDB ID: 2DJM) by PyMOL. **B)** The MoApx1-His fusion proteins were examined for the ability to co-sediment with total starch. The empty-His proteins were used as control. The supernatant (S) and pellet (P) fractions were probed with an anti-His antibody. **C and D)** Biomembrane interferometry was employed to evaluate the binding capacity of MoApx1 with cyclodextrins (CDs). The sensorgrams display real-time binding responses, with different concentrations of α-CD and β-CD injected over immobilized MoApx1. The resulting curves indicate the binding kinetics, allowing for the calculation of association and dissociation rates. **E and F)** Microscale thermophoresis (MST) was employed to evaluate the binding capacity of MoApx1 with CDs. Ten-micrometer MoApx1-His or empty-His was labeled by RED-NHS. The raw data were integrated and fitted to a binding model using the MST analysis software. The recombinant proteins were contained in NT standard capillaries. The solid curve is the fit of the data points to the standard Kd-fit function. Kd, dissociation constant. Each binding assay was repeated 3 times independently (*n* = 3), and the bars represent Sd. **G)** Natural mutations in the SBD domain and the SBD deletion mutant showed significantly reduced α-CD binding abilities by MST assay. The mean values of 3 measurements with Sds are shown.

During the examination of the MoApx1–OsPsaD interaction model, we discovered that MoApx1 primarily interacts with OsPsaD through its APX domain. However, there are also 2 unknown β-sheet-rich structures at its C-terminus (Fig. 6A). Screening of the PDB database for these structures revealed a strong resemblance to the known starch-binding domain (SBD) of a *Rhizopus oryzae* protein (PDB ID: 2DJM). We named these 2 structures SBD1 (307 to 426 aa) and SBD2 (427 to 548 aa) (Fig. 6A). Intriguingly, MoApx1 is notably conserved in Ascomycetous fungi, where it typically features both the APX and SBD structures. In contrast, its homologs in plants, bacteria, oomycetes, and some nonpathogenic fungi are primarily conserved only in the APX domain, lacking the C-terminal SBD domain (Supplementary Fig. S10). Despite the structural similarity in the SBD domain, overall sequence similarity remains relatively low (Supplementary Fig. S11).

To determine whether MoApx1 binds to starch directly, we conducted an in vitro glucan-binding assay. As a control, we used non-starch substance Sephadex G-10 beads to exclude nonspecific binding or protein precipitates (David et al. 2022; Dong et al. 2024; Yan et al. 2024), and the results showed that the recombinant MoApx1-His protein, but not free His protein, was able to bind to both rice leaf starch and maize soluble starch (Fig. 6B). Starch degradation results in the production of a mixture of linear and branched maltooligosaccharides (MOS), including cyclodextrins (CDs) (Biwer et al. 2002). To determine MOS that MoApx1 binds, we conducted a biolayer interferometry assay using glucose, maltose, maltotriose, maltotetraose, maltopentaose, α-CD (maltohexaose), β-CD (maltoheptaose), and γ-CD (maltooctaose). The results showed that, compared to other MOS, α-CD, and β-CD exhibit a strong binding affinity to MoApx1, demonstrating a concentration-dependent interaction (Fig. 6, C and D, and Supplementary Fig. S12). This result was further validated by using a microscale thermophoresis (MST) assay that revealed a binding constant of 0.6 *µ*m with α-CD and 1.9 *µ*m with β-CD (Fig. 6, E and F). This finding suggested that MoApx1 may have a higher affinity for MOS with a higher degree of polymerization produced by starch hydrolysis.

Notably, 3 natural mutations (E397Q, A407T, and P466L) in the SBD domain and the SBD deletion mutant showed significantly reduced α-CD binding ability and virulence, compared to full-length MoApx1 and A394S mutation (Fig. 6G and Supplementary Fig. S3, A to D). DPI treatment did not significantly increase the virulence of different SBD mutants (Supplementary Fig. S3E), indicating that IH growth regulated by the SBD domain is independent of ROS.

**Figure 7.**
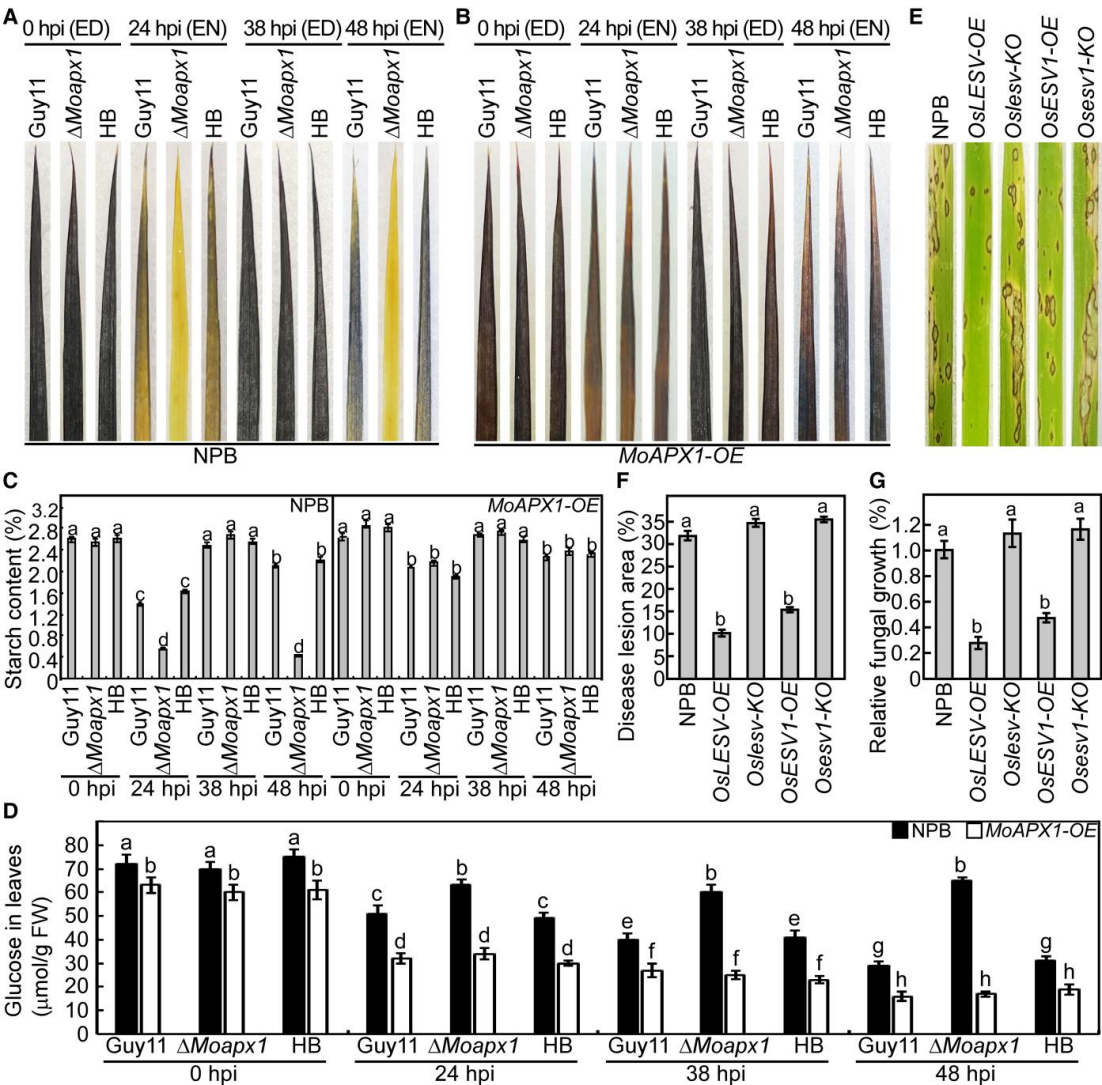
MoApx1 inhibits starch-mediated resistance against *M. oryzae*. **A and B)** Iodine staining of the leaves of NPB and *MoAPX1-OE* rice plants inoculated with Guy11 strain at different infectious stages. ED represents the end of the day, EN represents the end of the night, and HB represents the complemented strain (Δ*Moapx1*/*MoAPX1*). **C)** The contents of total starch were determined in leaves of NPB and *MoAPX1*-*OE* rice plants inoculated with the Guy11 strain at different infectious stages. The mean values of 3 measurements with Sds are shown. Significant differences were determined by 2-sided Duncan's new multiple-range tests, which were marked with different letters (*P* < 0.05). **D)** The determination of glucose in rice leaves. The glucose content was measured in the leaves of NPB and *MoAPX1-OE* rice lines at 0, 24, 38, and 48 h following inoculation with the Guy11 strain, the Δ*Moapx1* mutant, and the complemented strain. The mean values of 3 measurements with Sds are shown. Significant differences were determined by 2-sided Duncan's new multiple-range tests, which were marked with different letters (*P* < 0.05). **E to G)** The resistant phenotype of *Oslesv-KO*, *OsLESV-OX*, *Osesv1-KO*, and *OsESV1-OX* lines against *M. oryzae* the conidial suspension spray assay. Photos were taken at 7 dpi. The disease lesion area was assessed using ImageJ software, and the fungal growth was measured by quantifying *M. oryzae* genomic 28S rDNA relative to rice genomic Rubq1 DNA. The mean values of 3 determinations with Sds are shown. Significant differences were determined by 2-sided Duncan's new multiple-range tests, which were marked with different letters (*P* < 0.01).

To further confirm that MoApx1 binds to starch in vivo, we stained NPB and *MoAPX1*-*OE* rice lines at different infection stages using iodine solution. The stages include 0 and 38 hpi at the end of the day (ED) and 24 and 48 hpi at the end of the night (EN). We observed significant starch accumulation in leaves at ED, regardless of fungal strains or rice varieties. In contrast, we noted starch degradation in leaves inoculated with the Δ*Moapx1* mutant at EN compared to controls (Fig. 7A). Notably, this degradation was blocked in the *MoAPX1*-*OE* rice lines (Fig. 7B). We further measured the starch content and obtained results that are consistent with the staining observations (Fig. 7C). These results indicated that MoApx1 binds to starch and inhibits starch degradation during the *M. oryzae*–rice interaction.

The final product of starch hydrolysis is glucose, a critical energy source for plants (Thalmann and Santelia 2017; Ribeiro et al. 2022). To further investigate whether starch hydrolysis influences glucose levels in plants, we measured the glucose content in the leaves of wild-type NPB and *MoAPX1-OE* rice lines at various time points following inoculation with Guy11, the Δ*Moapx1* mutant, and the complemented strain. The results indicated that in NPB plants, infection by Guy11 and the complemented strain resulted in a continuous and significant decline in glucose contents. In contrast, the Δ*Moapx1* mutant only caused a significant decrease during the early stages of infection (0 to 24 h), with no changes observed at 24, 38, and 48 hpi. In the *MoAPX1-OE* line, infection by the different strains led to a more pronounced decrease in glucose content compared to NPB, and no significant difference was observed between the strain themselves (Fig. 7D).

To further confirm the relationship between starch synthesis in rice and resistance to *M. oryzae* infection, we analyzed the resistance of transgenic rice lines expressing 2 key starch synthesis proteins, OsLESV and OsESV1 (Dong et al. 2024). We found that the overexpression lines (*OsLESV*-*OE* and *OsESV1*-*OE*) showed enhanced resistance compared to NPB, while the knockout lines (*Oslesv*-*KO* and *Osesv1*-*KO*) exhibited increased susceptibility (Fig. 7, E to G). This finding suggested that starch accumulation in rice is critical for its resistance to the blast fungus.

## Discussion

In the interaction between plants and pathogens, hosts utilize pattern recognition receptors (PRRs) to perceive pathogen-associated molecular patterns (PAMPs) released by the pathogens, activating PAMP-triggered immunity (PTI), which is often accompanied by ROS burst (Jones and Dangl 2006). Chloroplasts are one of the main sites for ROS production. During PTI, the ROS generated in chloroplasts helps to enhance the plant's defense (Kachroo et al. 2021). In such oxidative environments, ROS-degrading enzymes could play critical roles in affecting pathogen survival. In fact, catalase-deficient mutants of *Claviceps purpurea*, *Botrytis cinerea*, and *Cochliobolus heterostrophus* all showed increased sensitivity to H_2_O_2_. However, none of these mutants showed altered pathogenicity (Garre et al. 1998; Schouten et al. 2002; Robbertse et al. 2003). This indicates that simply eliminating ROS is insufficient to facilitate pathogen infection, and additional factors are involved in the process.

**Figure 8.**
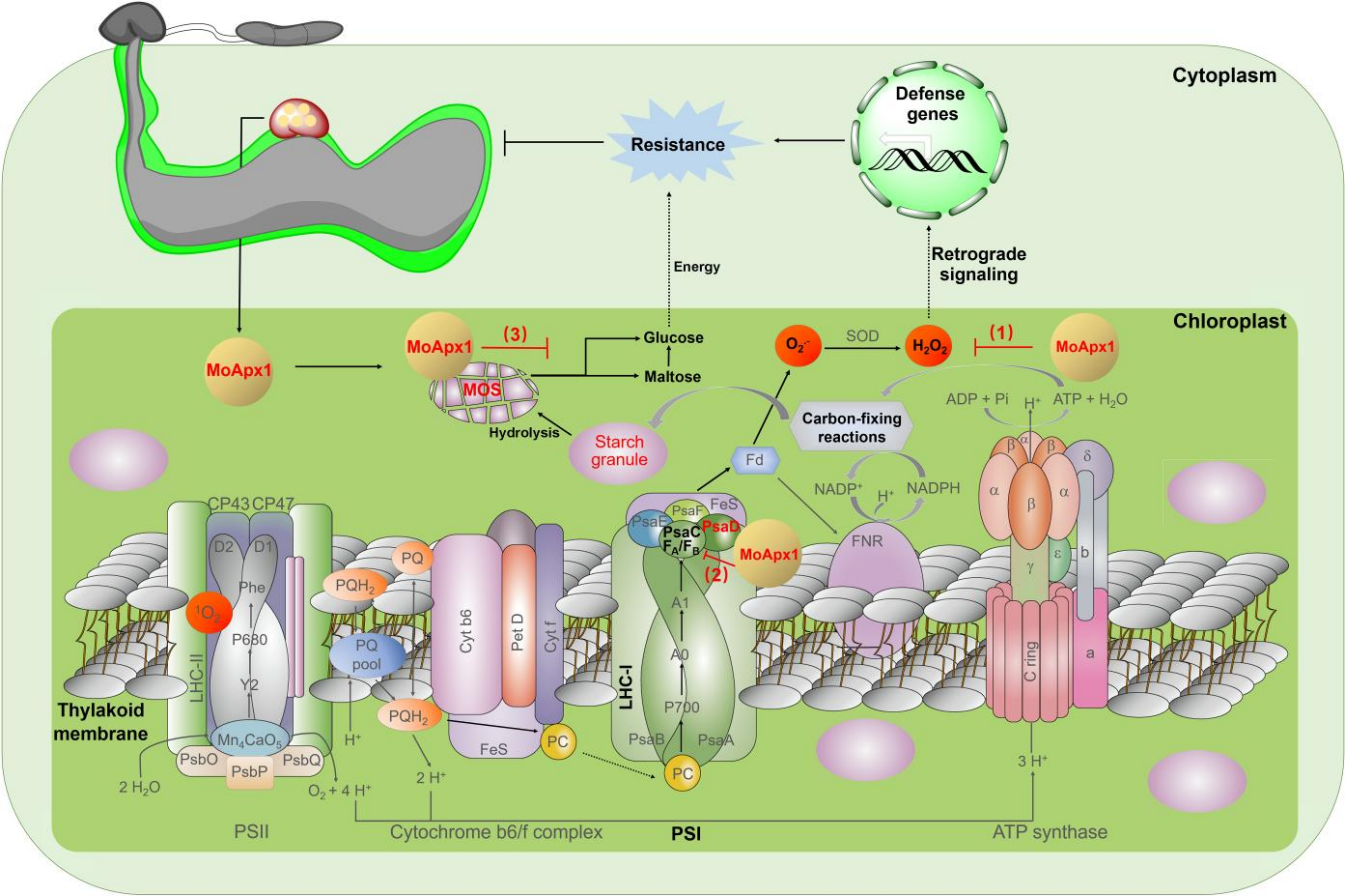
A proposed model of MoApx1 functioning to suppress host immunity. There are 3 roles of the ascorbate oxidase MoApx1 during the *M. oryzae*–rice interaction. (1) MoApx1 secreted by the rice blast fungus functions as an extracellular peroxidase to neutralize the host's H_2_O_2_ directly. (2) MoApx1 targets the PSI subunit OsPsaD, which is crucial for host immunity, thereby inhibiting cROS bursts. (3) MoApx1 features a starch-binding domain that effectively binds to the host's starch, potentially preventing its degradation and thereby disrupting the plant's energy supply and resistance to the disease.

For successful colonization, pathogens secrete numerous effector proteins that target and interfere with host immunity, thereby compromising PTI (Wang and Wang 2018; Zhang et al. 2024). It remains unclear whether these ROS-degrading enzymes function as effectors that interfere with host immunity. Here, we demonstrated that *M. oryzae* ascorbate oxidase MoApx1 functions as a multifunctional extracellular enzyme in the host. Its peroxidase activity directly neutralizes H_2_O_2_ in the host. It targets the PSI subunit OsPsaD to inhibit cROS bursts. Moreover, its starch-binding domain binds to starch in the host, preventing its degradation and potentially disrupting the energy supply, thus preventing resistance to the infection (Fig. 8).

Research on APX is currently focused mainly on plants and mammals. In plants, distinct APX isoforms can occur in multiple subcellular compartments, including chloroplasts, mitochondria, peroxisomes, and the cytosol, where it modulates cellular levels of H_2_O_2_ (Li 2023). It is well established that APXs play crucial roles in protecting plant cells against diverse environmental stresses, including pathogen attack. For example, *Vitis vinifera* ascorbate peroxidase VvApx1 promotes plant resistance to the Oomycete pathogen *Plasmopara viticola* (Lv et al. 2023). In maize, the overexpression of ZmApx1 results in lower H_2_O_2_ accumulation is accompanied by an accumulation of JA that enhances resistance against *Bipolaris maydis* (Zhang et al. 2022). In *Puccinia striiformis* f. sp. *tritici*–wheat interaction, thylakoid-associated ascorbate peroxidase (tAPX) phosphorylated by the wheat stripe rust resistance protein (WKS1) reduces the ability of the cells to detoxify ROS and contributes to resistance against the rust fungus (Gou et al. 2015). However, research on APX in fungi has primarily focused on its enzymatic activity (Schouten et al. 2002; Tanabe et al. 2011; Maruta et al. 2016), while its role in suppressing host immunity is still not well understood.

We have shown that MoApx1 is an extracellular enzyme secreted through the BIC structure that can enter rice cells rather than being retained in the apoplast (Khang et al. 2010; Zhang and Xu 2014). We demonstrated that MoApx1 enzymatic activities are important for the virulence of *M. oryzae*. We also found 2 key enzymatic activity sites and identified site-specific mutations among natural populations that significantly attenuate the virulence of *M. oryzae*. This is similar to the evolutionary pattern observed in ascorbate oxidase MoAo1 in *M. oryzae* (Hu et al. 2022).

Previous studies suggested that extracellular redox enzymes mainly inhibit the host's ROS through their enzymatic activities (Schouten et al. 2002; Robbertse et al. 2003; Tanabe et al. 2011; Li et al. 2015). In addition to the enzymatic activity, little is known about whether these enzymes also target other critical components of the host immune system to suppress its function. MoAo1 targets and inhibits the activity of rice OsAO3 and OsAO4, which regulates the apoplast redox status and plant immunity. This modulation of the apoplast redox status in rice cells plays a crucial role in virulence (Hu et al. 2022). Studies of MoApx1 revealed that it not only enhances pathogen virulence through its enzymatic activity but also targets the host PSI subunit OsPsaD to inhibit photosynthesis. By interfering with electron transport efficiency, MoApx1 suppresses the burst of cROS, thereby inhibiting host immunity.

The photosynthetic electron transport chains, including PSI and PSII, are the primary processes responsible for cROS production and host immunity (Kretschmer et al. 2019). Pathogens have evolved effectors to suppress this process, acknowledging the crucial role of photosystems in immunity. For example, *Pyrenophora tritici*-*repentis* secretes ToxA that targets the chloroplast ToxA Binding Protein 1 (ToxABP1/Thf1/Psb29 putative ortholog) to induce ROS accumulation through decreasing PSI and PSII abundance (Manning et al. 2009; Manning et al. 2010; Beckova et al. 2017). *P. syringae* secretes the cysteine protease HopNI that cleaves PsbQ from PSII to reduce oxygen production, electron transport, and the accumulation of cROS (Rodriguez-Herva et al. 2012). Similar to Psb29 and PsbQ in disease resistance, OsPsaD positively regulates the resistance to rice blast. Notably, both Psb29 and PsbQ are the subunits of PSII, the effector target PSI has not been reported. Unlike Hop1, which degrades PsbQ through its protease activity, MoApx1 does not affect the stability of OsPsaD protein. Instead, it mainly competes with OsPsaC for OsPsaD binding, which represents a new pathway for the pathogen that attacks the host.

Structure analysis revealed that MoApx1 contains a starch-binding domain, which is conserved among homologous proteins in fungi but not those of plants, oomycetes, bacteria, or mammals. Proteins containing the starch-binding domain include α-amylase, glucoamylases, carbohydrate-binding modules, and glycoside hydrolase family proteins (Norouzian et al. 2006; Haddad Momeni et al. 2019; Farooq et al. 2021; Xia et al. 2021), and the domain's function in fungal virulence remains not characterized. We showed that the starch-binding domain of MoApx1 enables its binding to leaf starch and cyclodextrins. The latter can trigger the biosynthesis of secondary metabolites and PR proteins that activate host immunity (Bru et al. 2006; Sabater-Jara et al. 2014; Pankaew et al. 2023). Therefore, the binding of MoApx1 to cyclodextrins could inhibit their ability to trigger host immune responses.

Additionally, stored starch serves as a crucial energy source required for the plant stress response when immediate glucose is exhausted (Thalmann and Santelia 2017; Ribeiro et al. 2022). The binding of MoApx1 to starch can inhibit rice's utilization of starch, potentially disrupting the energy supply. Notably, we found that starch synthesis in leaves critical for blast resistance. Despite mechanisms by which MoApx1 regulates fungal virulence and modulates rice immunity, our studies of MoApx1, its binding to host starch, and mutations in the starch-binding domain affecting virulence remain in early stages, and further studies are warranted.

## Materials and methods

### Fungal strains and cultures

*M. oryzae* wild-type strain Guy11 was used in this study. The strains were cultured in a complete medium (CM). Liquid CM was employed to cultivate mycelia for DNA and RNA extraction. For conidiation, strain blocks were maintained on a straw decoction and corn (SDC) agar medium at 28 °C for 7 d in the dark, followed by 3 d of continuous illumination under fluorescent light.

### Plant varieties

*OsLESV* and *OsESV1* overexpression and Crispr-Cas9 knockout transgenic rice plants were obtained from Xiangjin Wei of the China National Rice Research Institute, Hangzhou, China. *Oryza sativa japonica* cv. ‘Nipponbare’ was transformed with constructs pCAM2300:*MoAPX1*, pCAM2300:*OsPsaD*, and CRISPR-Cas9:*OsPsaD* with *Agrobacterium tumefaciens*-mediated transformation at Edgene Biot (Wuhan, China). The plants were grown in the greenhouse or fields at Nanjing Agricultural University in Nanjing, Jiangsu, China.

### Target gene deletion and complementation

The *MoAPX1* gene deletion mutant was generated using the standard 1-step gene replacement strategy (Zhang et al. 2011). Briefly, the fragments with 1.0 kb of sequences flanking the targeted genes were amplified by PCR with primer pairs. The resulting PCR products of target genes were digested with restriction endonucleases and inserted into the pCX62 vector with a hygromycin-resistance cassette (*HPH*) gene. The 3.4-kb fragment contains the flanking sequences, and the *HPH* cassette was amplified and introduced into Guy11 protoplasts. Putative transformants were verified by PCR and confirmed by Southern blotting analysis. The complement fragments, containing the native promoter region and entire coding region, were amplified by PCR and inserted into pYF11 (Thermo Fisher Scientific, R25001). The complementation vectors were transformed into mutant protoplasts and selected by bleomycin resistance. All primers used for gene deletion are listed in Supplementary Table S3.

### Mycological phenotype assay

The mycological phenotypes of *M. oryzae* strains were tested as described previously (Zhang et al. 2011). For vegetative growth, small agar blocks (2 × 2 mm) were cut from the edge of 4-d-old *M. oryzae* colonies and placed onto fresh CM media for culturing in the dark at 28 °C for 7 d. For conidia production, mycelia were grown in the dark on SDC or CM medium at 28 °C for 7 d, followed by constant illumination for 3 d. Measure the colony diameter before collecting the spores. Add 3 mL of sterile ddH_2_O to each plate. Use a glass rod or brush to spread the colonies. Filter through 1 layer of Miracloth and collect the spores in 10 mL or 50 mL tubes. Pipette 20 *µ*L of the spore suspension onto a hemocytometer and count the spores. Convert the results to A × 100 spores/cm^2^. Use statistical analysis to determine if there is a significant difference in conidial production between the mutant and wild-type strains. For conidium germination and appressorium formation, spore suspensions were centrifuged at 4,000 × *g*, washed 3 times with sterile ddH_2_O, and adjusted to 2 to 5 × 10^4^ spores/mL. Twenty microliters was pipetted onto a hydrophobic interface, with 2 to 3 inoculations per slide. Inoculated plants were incubated in a moisture-controlled incubator and kept in the dark at 28 °C. Germination and appressorium formation were observed.

### Virulence assay

For rice leaf spray assay, conidia were suspended to a concentration of 5 × 10^4^ spores/mL in a 0.2% (w/v) gelatin solution. Four milliliters of this suspension were sprayed on 12-d-old rice seedlings. The inoculated plants were then kept in a growth chamber at 25 °C with 90% humidity in the dark for the first 24 h, followed by a 16/8 h light/dark cycle. The disease severity was assessed at 7 d post-inoculation.

For rice leaf punch inoculation, conidia were suspended to a concentration of 1 × 10^5^ spores/mL in a 0.2% (w/v) gelatin solution. Four milliliters of this suspension was sprayed on 3-wk-old rice seedlings. Dip 10 *μ*L spore suspension for each drop using transferpettor at 2 spots on each leaf. Inoculated plants were incubated in a moisture-controlled incubator and kept dark at 28 °C for 24 h, followed by alternate light and dark cycles. The mean lesion size was assessed at 7 d post-inoculation.

### Invasive hyphae growth assay and ROS observation

For penetration and invasive growth assessments, conidial suspensions (1 × 10^5^ spores/mL) of Guy11, mutants, and the complemented strains were injected into rice leaf sheaths. The inner epidermis of infected sheaths was harvested at different hours post-inoculation and observed under a light microscope. Statistics of invasive hyphal growth at ∼100 appressorial penetration sites by rating the hyphal growth from levels I to IV (I, appressoria without primary invasive hypha; II, with primary invasive hypha; III, secondary invasive hypha does not extend to the neighboring plant cells; IV, invasive hypha extended into neighboring plant cells). The fluorescence observations were made using LSM980 ZEN 3.3 (Zeiss, Oberkochen, Germany).

To observe ROS, rice leaves or sheaths were stained with DAB (Sigma-Aldrich), as described previously (Liu et al. 2024). At 24 and 48 hpi, inoculated sheaths were incubated in 1 mg/mL DAB solution, pH 3.8, at room temperature for 8 h. Following destaining with a clearing solution (ethanol:acetic acid = 94:4, v/v) for 1 h, the inner epidermises were harvested and observed under a light microscope.

### Y2H assay

*MoAPX1* cDNA was cloned into the pGADT7 bait vector and the prey construct was generated by cloning *OsPsaD* or *OsPsaC* cDNA into the pGBKT7 vector. Both constructs were confirmed by sequencing analysis and subsequently transformed into the yeast strain AH109. Tryptophan (Trp+) and leucine (Leu+) transformants were isolated and assayed for growth on SD-Trp-Leu-His (histidine)-Ade (adenine) medium. The primers for Y2H assay are listed in Supplementary Table S3.

### In vivo co-IP assay

Co-IP using rice protoplast cells was performed as previously described (Liu et al. 2019). Briefly, *MoAPX* (without signal peptide sequence) and *OsPsaD* coding sequences, respectively, fused with GFP and RFP genes, were ampliﬁed and cloned into pCAM2300. Both plasmids were transformed into rice protoplasts via PEG-mediated transformation. Protoplasts were enriched by centrifugation, and proteins were extracted with IP buffer (50 mm Tris-HCl, pH 7.5; 150 mm NaCl; 0.5% NP-40; 5 mm DTT; and protease inhibitor cocktail). The supernatants were captured by adding 50 *μ*L GFP-Trap (ChromoTek, Germany), followed by shaking at 4 °C for an additional hour. The beads were recovered by centrifugation at 2,500 × *g* for 30 s and washed 6 times with cold TBS (50 mm Tris-HCl, pH 7.5; 150 mm NaCl). Then, 50 *μ*L of glycine elution buffer (pH 2.5) was added to the beads. After boiling for 5 min, the samples were loaded onto SDS-PAGE gels for Western blot analysis and detected using anti-GFP (M20004, Abmart, 1:5,000 dilution) and anti-RFP (5f8-100, ChromoTek, 1:3,000 dilution) antibodies. The primers for co-IP assay are listed in Supplementary Table S3.

### In vivo BiFC assay and subcellular localization

For subcellular localization in rice protoplasts, *MoAPX1* cDNA without the signal peptide was cloned into pCAM2300 to generate pACT1:*MoAPX1*^ΔSP^. MoApx1-nYFP and OsPsaD-cYFP fusion constructs were generated in pICH86988. pICH86988-nYFP and pICH86988-cYFP vectors were used as controls. The fusion constructs were transformed into protoplasts prepared from NPB seedlings, following a previously described method (Liu et al. 2024). Fluorescence in rice protoplasts was observed under a confocal microscope as above. The primers for Bimolecular Fluorescence Complementation (BiFC) assay are listed in Supplementary Table S3.

### In vitro pull-down assay

The RFP-tagged OsPsaD and HA-tagged OsPsaC were expressed and purified from rice protoplasts. To construct GST fusion plasmids, *MoAPX1* was inserted into pGEX4T-2 (GE Healthcare Life Science). GST-MoApx1 was expressed in *E. coli* strain BL21 (DE3) (Sigma, CMC0014) and purified as described (Yin et al. 2019). The pull-down assay was also carried out as described previously (Yin et al. 2019). Briefly, the GST, GFP, and RFP-fusion proteins were incubated with 50 *μ*L RFP beads (ChromoTek, Germany) for 2 h at 4 °C. After washing the beads 6 more times, the presence of fusion proteins was detected by immunoblotting using anti-GST (M20007L, Abmart), anti-HA (AT0024, Engibody), and anti-RFP antibodies. The primers for pull-down assay are listed in Supplementary Table S3.

### The O_2_.⁻ content and assay

The content of O_2_·⁻ was determined using a Superoxide Anion Content Assay Kit (Boxbio, China) with minor modifications. The standard solution was diluted to 0.4, 0.3, 0.2, 0.1, 0.05, and 0.025 *µ*mol/mL. For each standard, 40 *µ*L diluted solution was mixed with 60 *µ*L of extraction solution and 80 *µ*L of Reagent 1. Once incubated at 37 °C for 20 min, 60 *µ*L each of Reagents 2 and 3 was added and mixed, followed by incubating at 37 °C for another 20 min. Reactions were terminated by adding 100 *µ*L of chloroform. Once centrifuged at 8,000 × *g* for 5 min, 200 *µ*L of the upper aqueous phase were transferred to a 96-well plate and measurements were made using a microplate reader.

For sample preparation, protoplasts were adjusted to 5 × 10^6^ cells using a hemocytometer and treated with Flg22 for 10 min in the dark. One mL of extraction solution was then added, mixed, and centrifuged at 4 °C for 20 min. Forty-microliter extract solution was then withdrawn and mixed with extraction solution, Reagents 1, 2, and 3 as described above for establishing the standard curve. The superoxide anion content is expressed as *µ*mol/mL and the release rate as *µ*mol/min/mL, as described by the assay kit manufacturer.

### Electronic transfer efficiency assay

Electronic transfer efficiency between 2 leaves was measured using a DUAL-PAM-100 instrument (Zeal Quest Scientific Technology, China) following the instructions provided by the instrument manufacturer. A profile of P700 + Fluo was first created and a slow kinetics interface in the incurve system was selected using the DualPAM software.

### Puriﬁcation of starch granules from rice leaves

The starch granules were extracted according to the established method (Dong et al. 2024; Yan et al. 2024). Briefly, the leaves of NPB were homogenized in an extraction buffer containing 0.5% (v/v) Triton X-100, 50 mm Tris-HCl (pH 8.0), and 0.2 mm EDTA using a mortar and pestle. The homogenate was then filtered through a 15-mm nylon mesh. The insoluble materials were resuspended for a second extraction. The starch granules were collected by centrifugation at 3,000 × *g* in 95% (v/v) Percoll (Sigma) for 20 min. Subsequently, the SGs were washed with 0.5% (w/v) SDS, water, and finally with 80% (v/v) ethanol before being freeze-dried under vacuum overnight.

### Biomembrane interferometry assay

MoApx1 (pET32a-*MoAPX1*-*HIS*) was expressed in *E. coli* and purified. The biolayer interferometry assay was performed as previously described using Octet Red 96 (Forté Bio) (Yang et al. 2022). MoApx1 protein was biotinylated by EZ-Link NHS-Biotin (ThermoFisher Scientific). Proteins (75 *μ*g/mL) were loaded into equilibrated streptavidin (SA) biosensors. Association–dissociation cycles were performed by alternating sensors into compound solution and assay buffer wells. The signals were analyzed by a double reference subtraction protocol and a 1:1 binding model was used to fit the association and dissociation rates. Equilibrium dissociation constant (KD) values were calculated from the ratio of Koff to Kon. Glucose (A501991), maltose (A604950), maltotriose (A419387), maltotetraose (A418949), maltopentaose (A413242), α-CD (A426606), β-CD (A422962), and γ-CD (A425458) were purchased from Sangon Biotech (Nanjing, China).

### MST analysis

Binding of recombinant MoApx1-His and His control proteins with compounds was measured by MST in a Monolith NT.Label Free (Nano Temper Technologies GMBH) instrument. The samples were loaded into the NT.Label Free standard capillaries and measured with 20% LED power and 40% MST power. The KD-fit function of the Nano Temper Analysis Software (Version 1.5.41) was used to fit the curve and calculate the value of the dissociation constant (Kd).

### Chloroplast isolation

Chloroplast isolation in rice leaves was performed as previously described (Liu et al. 2019). For isolating protoplasts, a razor blade was used to cut the stems and leaves into ∼0.5 mm strips. They were then placed into a petri dish containing 10 mL enzyme solution (1.5% cellulase and 0.3% macerozyme). The vacuum was applied for 1 h for the infiltration of the enzyme solution. Plants were incubated for about 4 h in the dark with gentle shaking (∼40 rpm) at room temperature and the solution was removed with a glass pipet. Ten-milliliter W5 medium was added and allowed gently swirl (80 rpm) for 1 h to release the protoplast. W5 protoplasts were filtered through a 35 *μ*m nylon mesh, transferred into an 8 mL glass vial, precipitated at 220 × *g* for 4 min. The isolated chloroplast was used for Western blot analysis with the PsaD antibody (Agrisera).

### Transmission electron microscopy

For immunoelectron microscopy, conidial suspensions (1.5 × 10^5^ spores/mL) of Guy11/MoApx1-GFP were sprayed onto rice leaves. After 48 h of incubation at 28 ℃, the infected leaves were cut to 3 mm^2^ sections. The sections were fixed with 0.5% (v/v) glutaraldehyde and 3% (v/v) paraformaldehyde in 10 × PBS for 2 h at room temperature. The fixed sections were dehydrated through 50%, 70%, 90%, 100%, 100% ethanol series at −20 °C. After dehydrated, the samples were embedded in LR gold resin (Bioscience). Polymerization was carried out at −20 °C under long ultraviolet irradiation for 72 h. Then, samples were sectioned on an ultramicrotome (LKB Nova) with a diamond knife and the ultrathin sections were then immunolabeled with anti-GFP as the primary antibody, and then treated with anti-mouse conjugated with 10 nm diameter gold particles (0.5 *μ*g/*μ*L; Abcam) as the secondary antibody. Finally, the samples were examined under the transmission electron microscope.

### Leaf starch staining and content determination

Starch staining and content determination in rice leaves were performed as previously described (Dong et al. 2024; Yan et al. 2024). Briefly, rice leaves infected by *M. oryzae* at various stages were collected at the end of the day and night, decolorized in 80% ethanol, and stained with Lugol solution (Sigma) for 30 min before observing amylose–iodine complex in starch. To quantify starch levels, leaves were ground in liquid nitrogen and dried at 80 °C to a constant weight. Starch and amylose contents were measured using the Megazyme starch (K-TSTA) and amylose (K-AMYL) determination kits (Wicklow, Ireland).

### Phylogenetic analyses

The protein sequences were obtained from NCBI databases. The sequence alignment was performed using ClustalW in MEG, followed by manual refinement of gaps and misaligned regions, particularly in conserved domains critical for plant gene families. Next, select the best-fit substitution model using AIC/BIC criteria. Proceed to phylogenetic tree construction via neighbor-joining, applying the chosen substitution model, setting gamma-distributed rates if indicated, and running 1,000 bootstrap replicates. For tree visualization, open the tree file in MEGA, and adjust branch styles, colors, and labels via Taxonomy/Group Info, ensuring bootstrap values are displayed. Export the final tree as a high-resolution image for publication. Alignments used for phylogenetic analysis are provided in Supplementary Files S1 and S2.

### Determination of glucose contents

The glucose content was determined using a Glucose Content Assay Kit (BC1870, SOLARBIO) following the manufacturer's instructions. Briefly, 0.1 g of rice leaves and 1 mL of distilled water were mixed and ground. The mixtures were placed in a boiling water bath for 10 min, centrifuged at 8,000 × *g* for 10 min, and supernatants were collected for measurements. The glucose content (*µ*mol/g fresh weight) is calculated as follows: glucose content (*µ*mol/g fresh weight) = 0.5 *µ*mol/mL × (A_sample − A_blank)/(A_standard − A_blank)/sample fresh weight (g/mL).

### Statistical analysis

Quantiﬁcation analysis on disease lesion areas, pathogen growth, conidiation, germination, appressoria formation, and the content of starch and O_2_·⁻ were conducted using Origin 2024b and DPS 7.05 version software. All values are presented as mean ± Sd, with exact *n* values provided in the figures or legends. Statistical significance was assessed using either Student's *t*-test or Duncan's multiple-range test. All data are provided in Supplementary Data Set S1.

### Accession numbers

Sequence data from this article can be found in the GenBank/EMBL data libraries under accession numbers: MGG_09398 (*MoAPX1*), LOC_Os08g44680 (*OsPSAD*), and LOC_Os06g46436 (*OsPSAC*).

## Supplementary Material

koaf146_Supplementary_Data

## Data Availability

The data underlying this article are available in the article and in its online supplementary material.

## Supplementary data

The following materials are available in the online version of this article.

**Supplementary Figure S1**. Transcriptional patterns of the *MoAPX1* and *OsPsaD* genes.

**Supplementary Figure S2**. Target deletion of the *MoAPX1* gene.

**Supplementary Figure S3**. The polymorphism variations of MoApx1 are necessary for its enzymatic activity and normal virulence.

**Supplementary Figure S4**. *OsPSAD* and *MoAPX1* transgenic rice plants do not affect growth and yield.

**Supplementary Figure S5**. The structure models of MoApx1, OsPsaD, and OsPsaC were predicted by AlphaFold 3.

**Supplementary Figure S6**. The polymorphism sites do not affect the interactions between MoApx1 and OsPsaD.

**Supplementary Figure S7**. MoApx1 does not interact with OsPsaC.

**Supplementary Figure S8**. OsPsaD overexpression transgenic lines do not induce the accumulation of ^1^O_2_.

**Supplementary Figure S9**. Determination of susceptibility of MoApx1 overexpression transgenic rice lines.

**Supplementary Figure S10**. The APX domain, but not the SBD domain, of MoApx1 proteins is conserved across various species.

**Supplementary Figure S11**. Sequence alignment of MoApx1 homologies in fungi.

**Supplementary Figure S12**. Evaluation of the binding capacity of MoApx1 with different MOS.

**Supplementary Table S1**. Comparison of mycological characteristic among strains.

**Supplementary Table S2**. Identification of MoApx1 interacting proteins by Y2H.

**Supplementary Table S3**. Oligonucleotide primers used in this study.

**Supplementary Data Set S1**. All the statistical analysis data.

**Supplementary File S1**. Sequence alignment of Apx1 proteins.

**Supplementary File S2**. Phylogenetic analyses of Apx1 proteins.

## Dive Curated Terms

The following phenotypic, genotypic, and functional terms are of significance to the work described in this paper:

SDC Gramene: AT2G17690
SDC Araport: AT2G17690

LHCB5 Gramene: Cre16.g673650
LHCB5 Araport: Cre16.g673650

starch CHEBI: CHEBI:28017

MOS Gramene: malto-oligosaccharide
MOS Araport: malto-oligosaccharide

NCBI Gramene: https://www.ncbi.nlm.nih.gov
NCBI Araport: https://www.ncbi.nlm.nih.gov
