# Supplementary material for: The multifunctional ascorbate peroxidase MoApx1 secreted by Magnaporthe oryzae mediates the suppression of rice immunity
Source: Plant Cell. 2025 Jun 11;37(7):koaf146. doi: 10.1093/plcell/koaf146 (PMC12231552; doi:10.1093/plcell/koaf146)
Supplement: koaf146_Supplementary_Data [file koaf146_supplementary_data.zip › Supplementary Data_06.04.pdf]

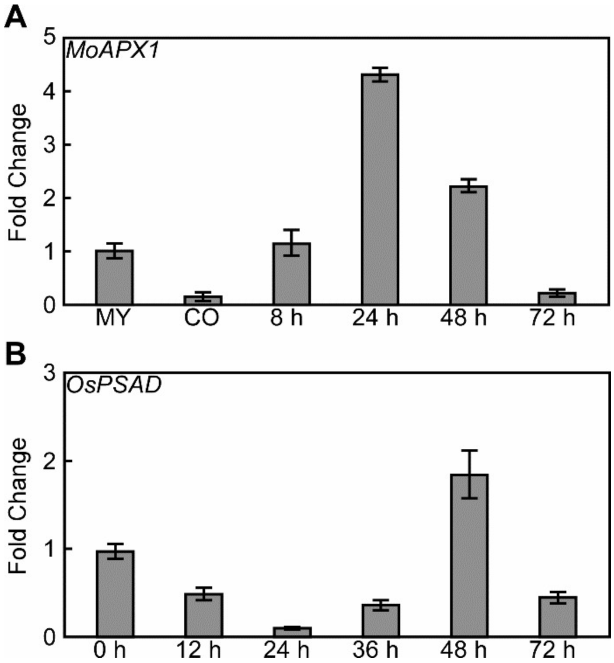

Supplementary Figure S1. Transcriptional patterns of the *MoAPX1* and *OsPsaD* genes. (A and B) Relative expression levels of *MoAPX1* and *OsPsaD* in infectious stages (8, 24, 36, 48, and 72 hpi) were determined by qPCR. MY represents mycelia, and CO represents conidia. Values are the means of three replications independently, and error bars represent SD (n=3).

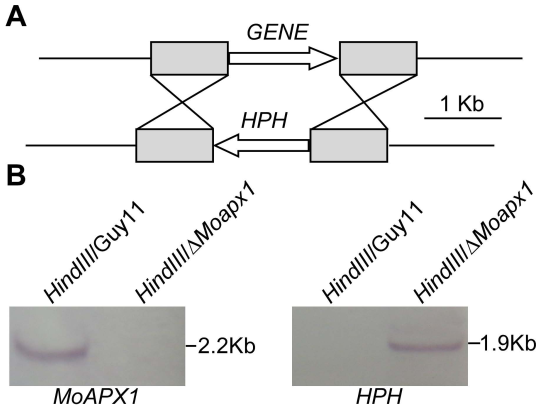

Supplementary Figure S2. Target deletion of the *MoAPX1* gene. (A) Southern hybridization was used to analyze the *MoAPX1* gene copy number and validate the deletion of *MoAPX1* and the addition of a single copy integration of the *HPH* gene in the Δ*Moapx1* mutants. **(B)** When hybridized with the specific *MoAPX1* gene probe, bands were observed in Guy11 but not in the Δ*Moapx1* mutant. When hybridized with the *HPH* probe, the Δ*Moapx1* mutant but not Guy11 exhibited specific bands characteristic of the gene replacement event.

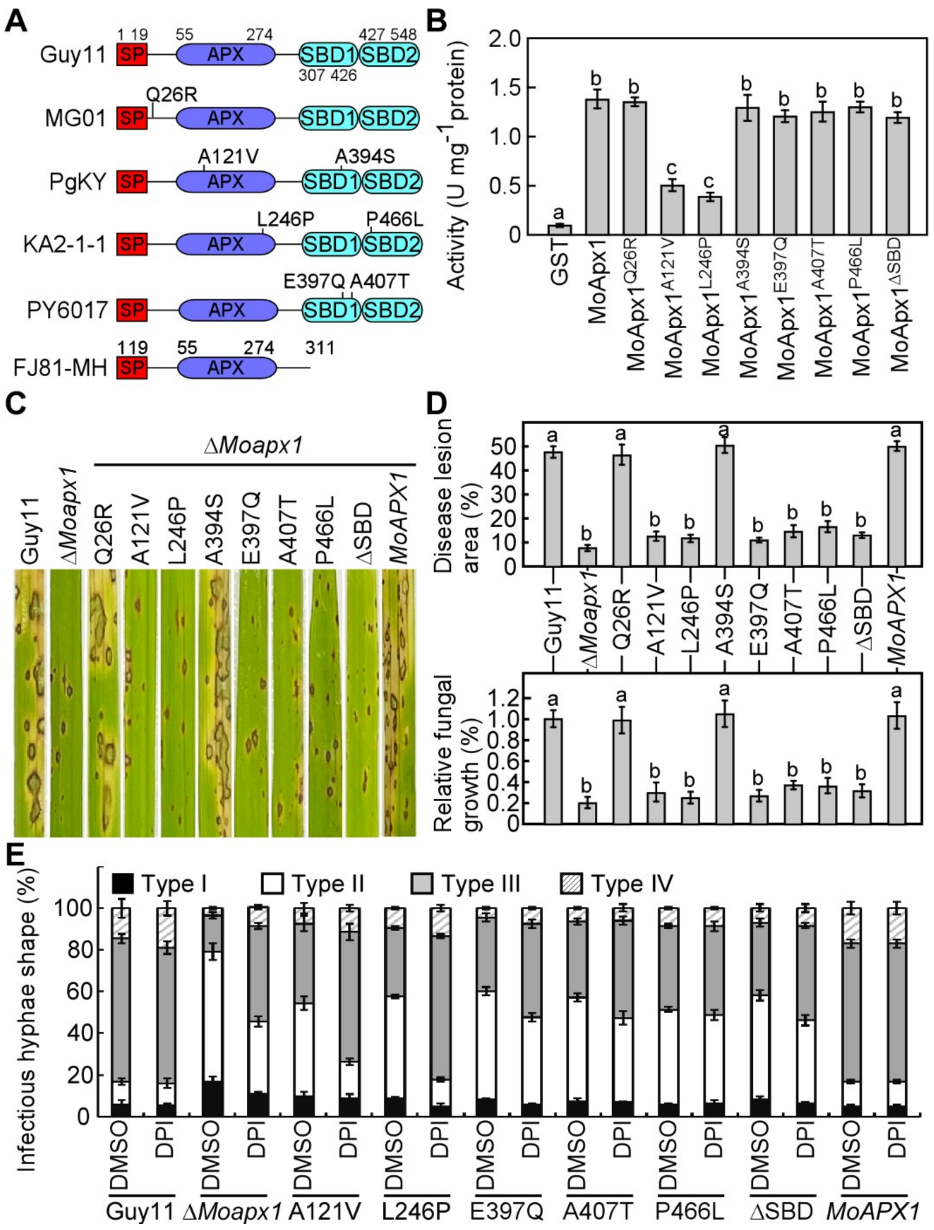

Supplementary Figure S3. The polymorphism variations of MoApx1 are necessary for its enzymatic activity and normal virulence. (A) Schematic drawings of MoApx1 from different *M. oryzae* isolates. The red box represents the signal peptide, the purple rectangles are the APX domains, and the blue rectangles are the SBD domains. The vertical curves are the polymorphic sites. (B) Detection of differenttypesofenzymaticactivitiesofMoApx1.PurifiedGST,GST-MoApx1, GST-MoApx1^{Q26R}, GST-MoApx1^{A121V}, GST-MoApx1^{L246P}, GST-MoApx1^{A394S}, GST-MoApx1^{E397Q}, GST-MoApx1^{A407T}, GST-MoApx1^{P466L}, and GST-MoApx1$^{\Delta SBD}$ proteins were added to the reactions. The reactions were monitored by the absorbance at 570 nm. Data represent observations from three independent experiments, and the error bars indicate the SD of three replicates. Significant differences were determined by two-sided Duncan's new multiple-range tests and marked with different letters ($p < 0.01$). (C and D) Pathogenicity of different mutation types of MoApx1 on rice using the conidial suspension spray assay. Diseased leaves were photographed at 7 dpi. The disease lesion area was assessed using ImageJ software. The mean values of 3 determinations with SDs are shown. Significant differences were determined by

two-sided Duncan's new multiple-range tests and marked with different letters ($p < 0.01$). (E) Invasive growth of the Δ*Moapx1* and its polymorphism sites mutants was restored by DPI treatment. Statistics of invasive hyphal growth at ~100 appressorial penetration sites by rating the hyphal growth from level I to IV. The experiments were repeated three times and showed similar results. Diphenyleneiodonium (DPI), a NADPH oxidase inhibitor, was dissolved in DMSO. The mean values of 3 determinations with SDs are shown.

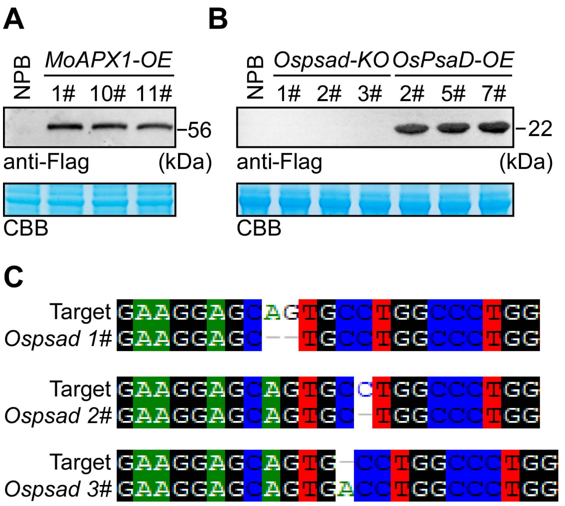

E

Lines	Height (cm)	TGW (g)	SSR (%)
NPB	99.32 ± 1.5	27.35 ± 0.7	68.31 ± 1.1
Ospsad -KO 1#	97.63 ± 1.4	28.42 ± 0.6	67.92 ± 1.3
Ospsad -KO 2#	96.83 ± 1.3	26.69 ± 0.8	68.03 ± 1.2
Ospsad -KO 3#	98.06 ± 1.1	26.87 ± 0.4	67.85 ± 1.3
OsPsaD -OE 2#	98.86 ± 1.1	27.02 ± 0.7	67.88 ± 1.1
OsPsaD -OE 5#	99.02 ± 1.2	28.10 ± 0.5	68.13 ± 1.2
OsPsaD -OE 7#	97.77 ± 1.3	27.08 ± 0.5	67.81 ± 1.1
MoAPX1 -OE 1#	98.23 ± 1.4	26.46 ± 0.6	68.03 ± 1.3
MoAPX1 -OE 10#	99.16 ± 1.6	27.49 ± 0.6	68.11 ± 1.3
MoAPX1 -OE 11#	97.92 ± 1.8	28.11 ± 0.4	67.72 ± 1.4

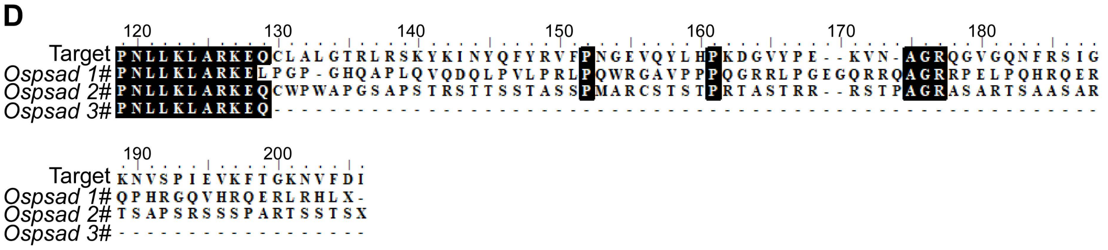

Supplementary Figure S4. *OsPSAD* and *MoAPX1* transgenic rice plants do not affect growth and yield. (A and B) Western blot assay for the protein level of transgenic MoApx1 and OsPsaD overexpression rice lines in the NPB background. Coomassie Brilliant Blue (CBB) was used as the loading control. (C) Target sites designed and verified for knocking out the *OsPSAD* gene by CRISPR/Cas9. The *Ospsad-KO* lines were verified by PCR-based sequencing. Three representative transgenic lines (#1, #2, and #3) are generated from NPB background. (D) The alignment of the amino acid sequence of *Ospsad-KO* lines with target. (E) The phenotype of *OsPSAD* and *MoAPX1* transgenic plants. The height, thousand-grain weights (TGW), and seed setting rate (SSR) were measured after the rice paddy matured.

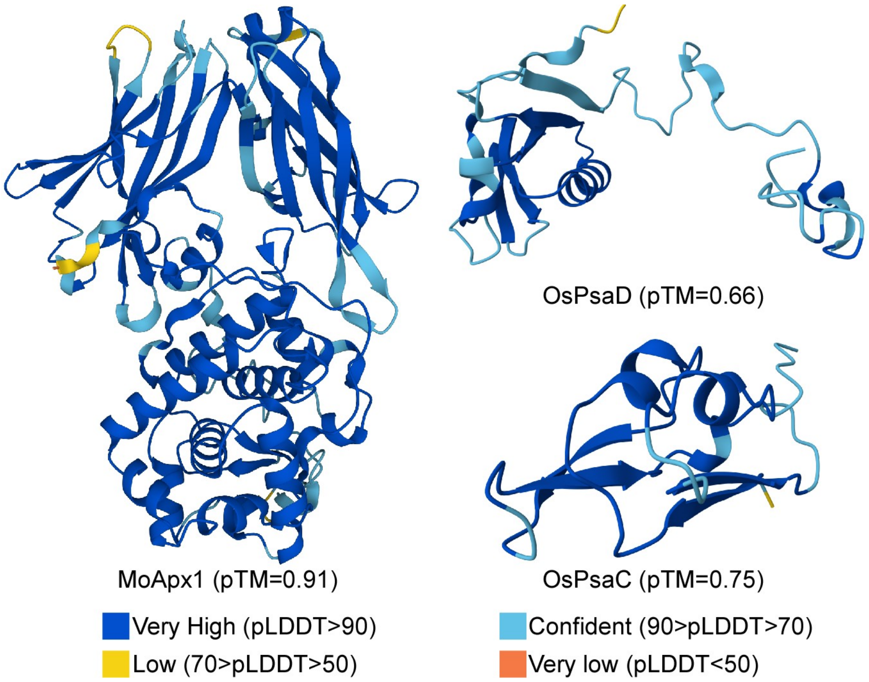

Supplementary Figure S5. The structure models of MoApx1, OsPsaD, and OsPsaC were predicted by AlphaFold3. AlphaFold produces a per-residue confidence score (pLDDT) between 0 and 100. The higher the score, the higher the credibility. A pTM score above 0.5 means the overall predicted fold for the complex might be similar to the true structure.

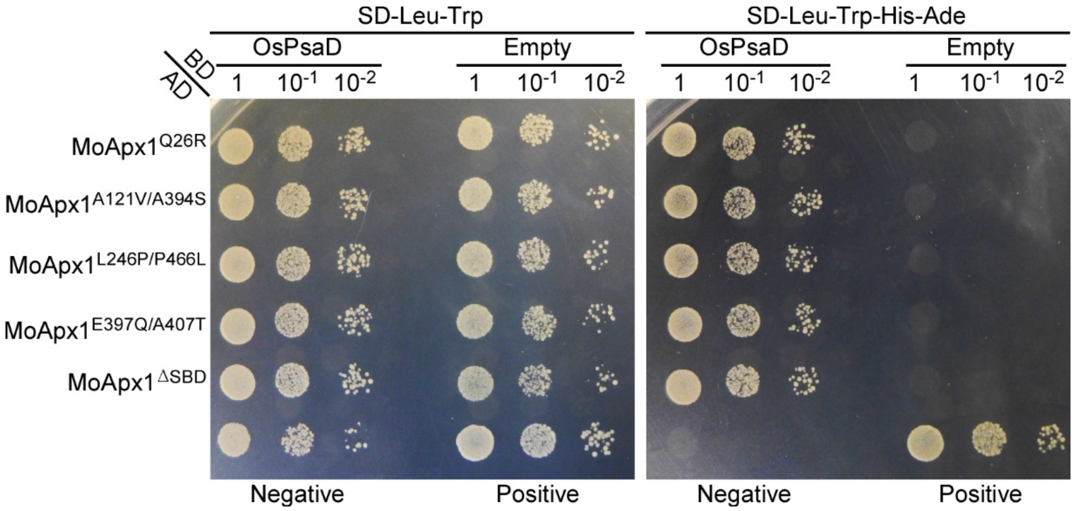

Supplementary Figure S6. The polymorphism sites do not affect the interactions between MoApx1 and OsPsaD. Yeast two-hybrid assay between AD-MoApx1 and its polymorphism sites mutations (without the signal peptide sequence) and BD-OsPsaD. Cells were plated on an SD-Leu-Trp medium and then transferred onto an SD-Ade-Leu-Trp-His medium.

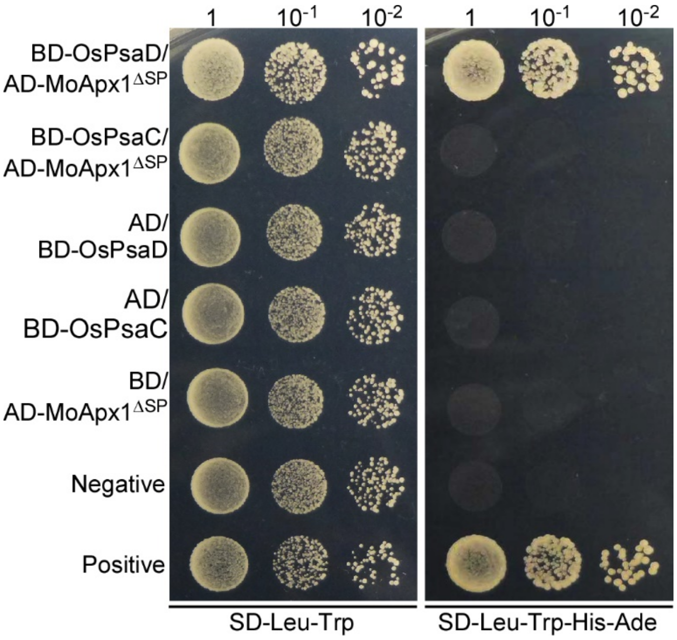

Supplementary Figure S7. MoApx1 does not interact with OsPsaC. Yeast two-hybrid assay between AD-MoApx1 (without the signal peptide sequence) and BD-OsPsaC. Cells were plated on a SD-Leu-Trp medium and then transferred onto SD-Ade-Leu-Trp-His medium.

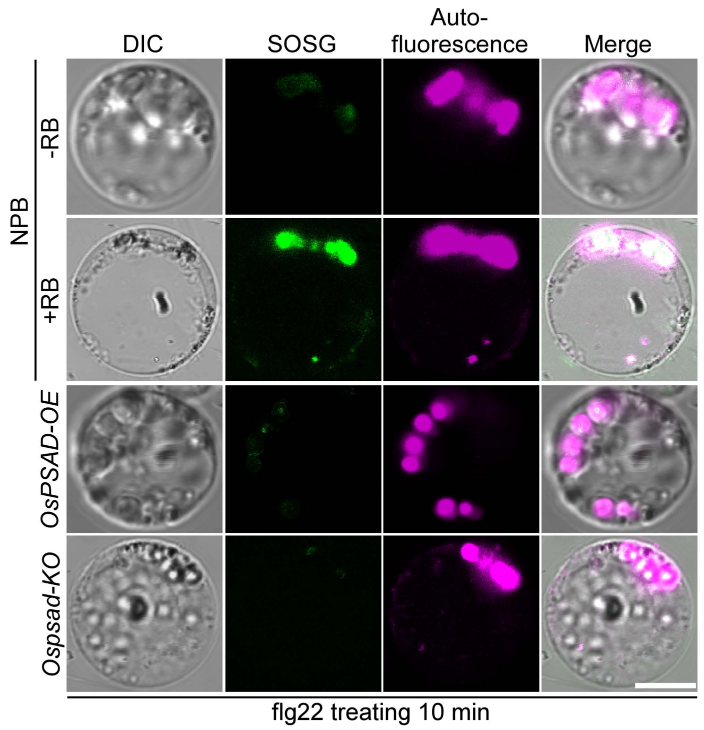

Supplementary Figure S8. OsPsaD overexpression transgenic lines does not induce the accumulation of $^1O_2$. The protoplast of NPB, *Ospsad*-*KO*, and *OsPsaD*-*OE* rice plants treated with 0.1 μM flg22 were stained with SOSG. The Rose Bengal (RB) can induce the production of $^1O_2$ in rice was used as a positive control. The red autofluorescence represents chloroplast. Scale bars, 5 μm.

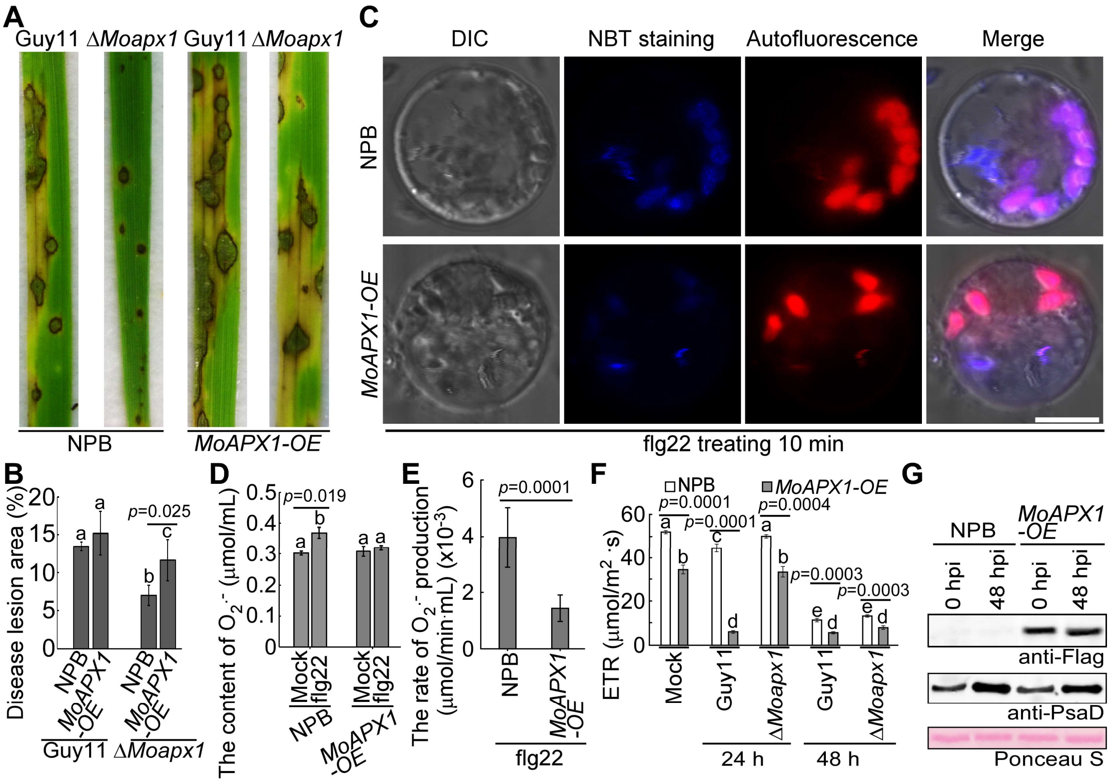

Supplementary Figure S9. Determination of susceptibility of MoApx1 overexpression transgenic rice lines. (A and B) Infection phenotype of *OsAPX1-OE* lines against *M. oryzae* using the conidial suspension spray assay. Photos were taken at 7 dpi. The disease lesion area was measured. The mean values of 3 measurements with SDs are shown. Significant differences were determined by two-sided Duncan's new multiple-range tests and marked with different letters ($p < 0.05$). (C) The *OsAPX1-OE* lines showed a lower accumulation of superoxide anions when treated with an elicitor. The NPB and *OsAPX1-OE* rice plants treated with flg22 were stained with NBT. The red autofluorescence represents chloroplast. (D and E) The determination of $O_2^{\cdot-}$. The content and production rate in the NPB and *OsAPX1-OE* rice plants when treated with or without flg22 were determined using Superoxide Anion Content Assay Kit. The mean values of 3 measurements with SDs are shown. Significant differences were determined by two-sided Duncan's new multiple-range tests and marked with different letters ($p < 0.05$). (F) The determination of ETR. The ETR in NPB and *OsAPX1-OE* rice plants infected by Guy11 and Δ*Moapx1* at 24 and 48 hpi was measured using the instrument DUAL-PAM-100. The mean values of 3 measurements with SDs are shown. Significant differences were determined by two-sided Duncan's new multiple-range tests and marked with different letters ($p < 0.01$). (G) The protein level of OsPsaD in NPB and *MoAPX1-OE* (Flag tagged MoApx1) rice lines inoculated with Guy11 at 48 hpi or without infection (0 hpi) was determined by immunoblotting using anti-PsaD polyclonal antibodies. Protein loadingisindicatedwithPonceaustaining.Scalebars,5μm.

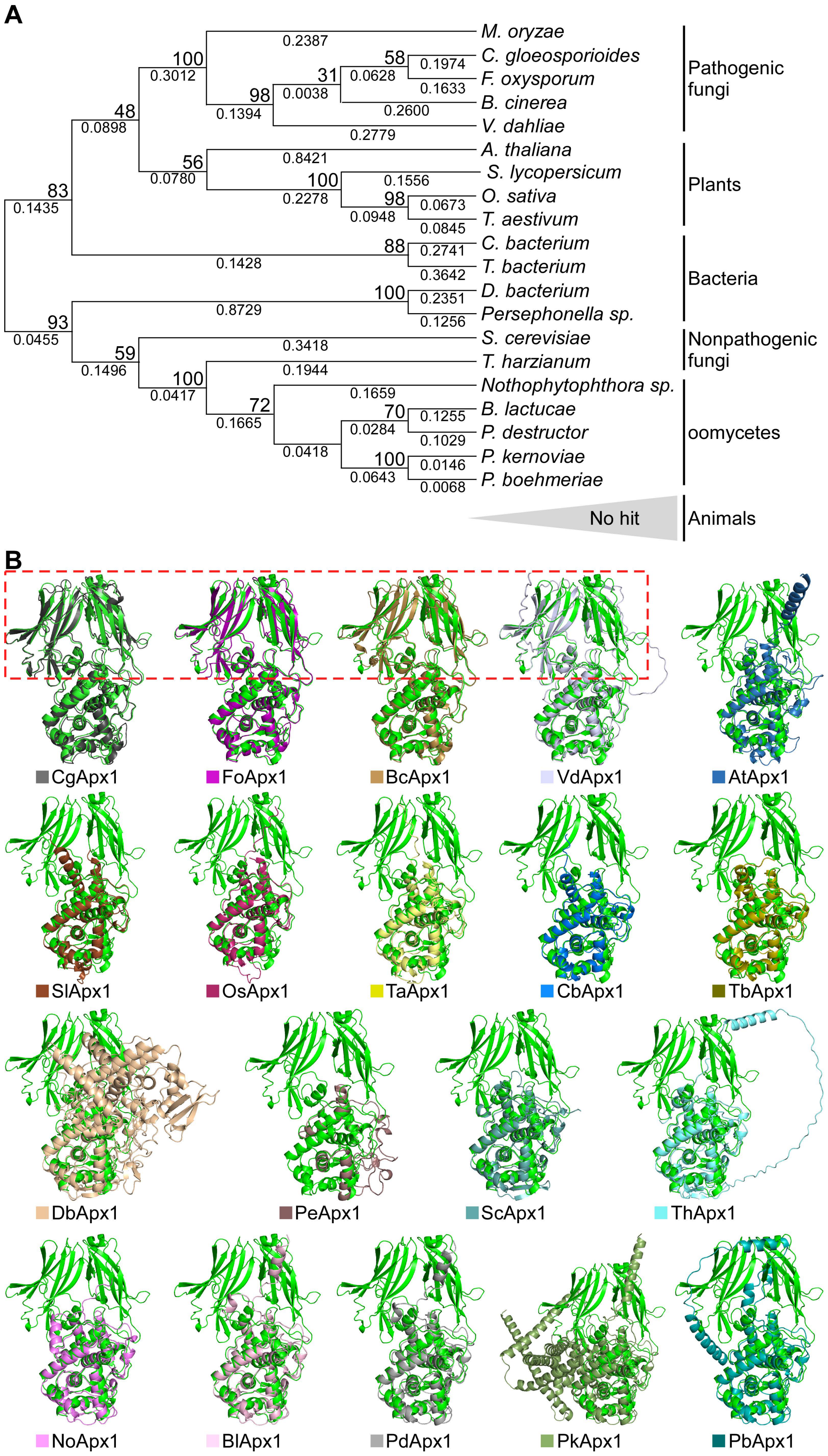

A
M. oryzae
C. gloeosporioides
F. oxysporum
B. cinerea
V. dahliae
A. thaliana
S. lycopersicum
O. sativa
T. aestivum
C. bacterium
T. bacterium
D. bacterium
Persephonella sp.
S. cerevisiae
T. harzianum
Nothophytophthora sp.
B. lactucae
P. destructor
P. kernoviae
P. boehmeriae
Pathogenic fungi
Plants
Bacteria
Nonpathogenic fungi
oomycetes
No hit
Animals
B
CgApx1
FoApx1
BcApx1
VdApx1
AtApx1
SlApx1
OsApx1
TaApx1
CbApx1
TbApx1
DbApx1
PeApx1
ScApx1
ThApx1
NoApx1
BlApx1
PdApx1
PkApx1
PbApx1

Supplementary Figure S10. The APX domain, but not the SBD domain, of MoApx1 proteins is conserved across various species. (A) The MoApx1 proteins from different species were aligned using the Clustal_W program, and the neighbor-joining tree was constructed with 1,000 bootstrap replicates by the use of MEGA 7.0. The sequences were obtained in the following organisms: *M. oryzae* (MGG_09398), *C. gloeosporioides* (ELA32452), *F. oxysporum* (FOXG_13788), *V._dahliae* (VDAG_03705), *B. cinerea* (G2Y2Z0_BOTF4), *T. harzianum* (A0A2T4AKQ6), *S. cerevisiae* (YKR066C), *A. thaliana* (AT5G05340.1), *S. lycopersicum* (A0A3Q7HLJ4), *O. sativa* (EAZ43377.1), *T. aestivum* (A0A3B6TID6), *Nothophytophthora* sp. *Chile5* (RLN98678.1), *B. lactucae* (TDH65662.1), *P. kernoviae* (KAG2530533.1), *P. boehmeriae* (KAG7400017.1), *C. bacterium* (HEY2594423.1), *T. bacterium* (HEY7030099.1), *D. bacterium* (MDT7943115.1), *L. bacterium* (A0A960Y2B1), *Persephonella* sp. (MDQ7056629.1). The identity of protein sequences was calculated by BioEdit. (B) Models of MoApx1 homologous proteins across different species were constructed using AlphaFold2. These models were colored in various hues and aligned with MoApx1 (green).

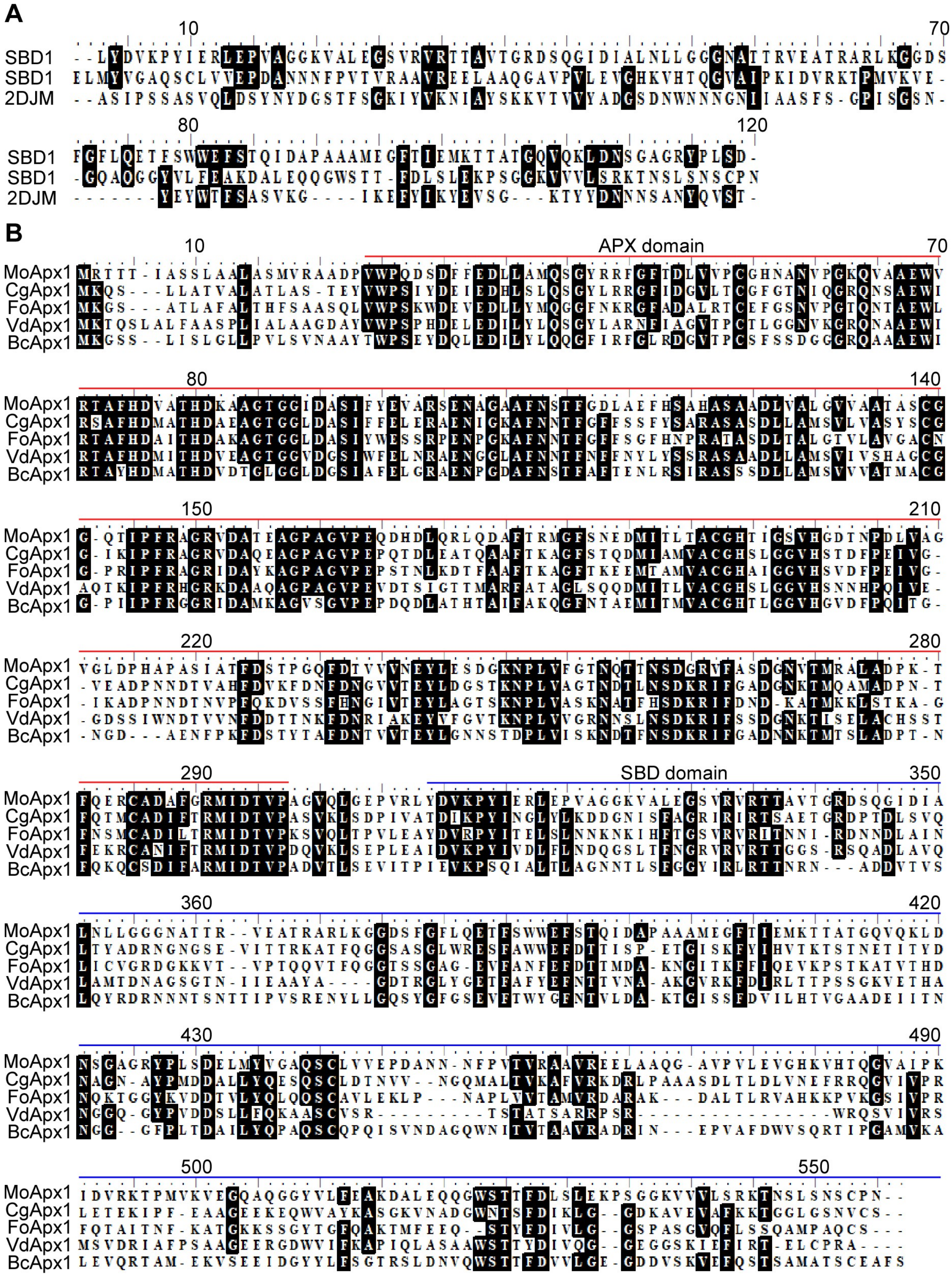

Supplementary Figure S11. Sequence alignment of MoApx1 homologies in fungi. (A) Comparison of the SBD domain of MoApx1 with the protein sequence of the SBD domain from *Rhizopus oryza*, which has a resolved crystal structure (PDB ID: 2DJM). (B) Protein sequence alignment of homologous proteins of MoApx1 in fungi. The sequences were obtained in the following organisms: *M. oryzae* (MGG_09398), *C. gloeosporioides* (ELA32452), *F. oxysporum* (FOXG_13788), *V._dahliae* (VDAG_03705), *B. cinerea* (G2Y2Z0_BOTF4). The APX domain is highlighted in red, and the SBD domain is highlighted in blue.

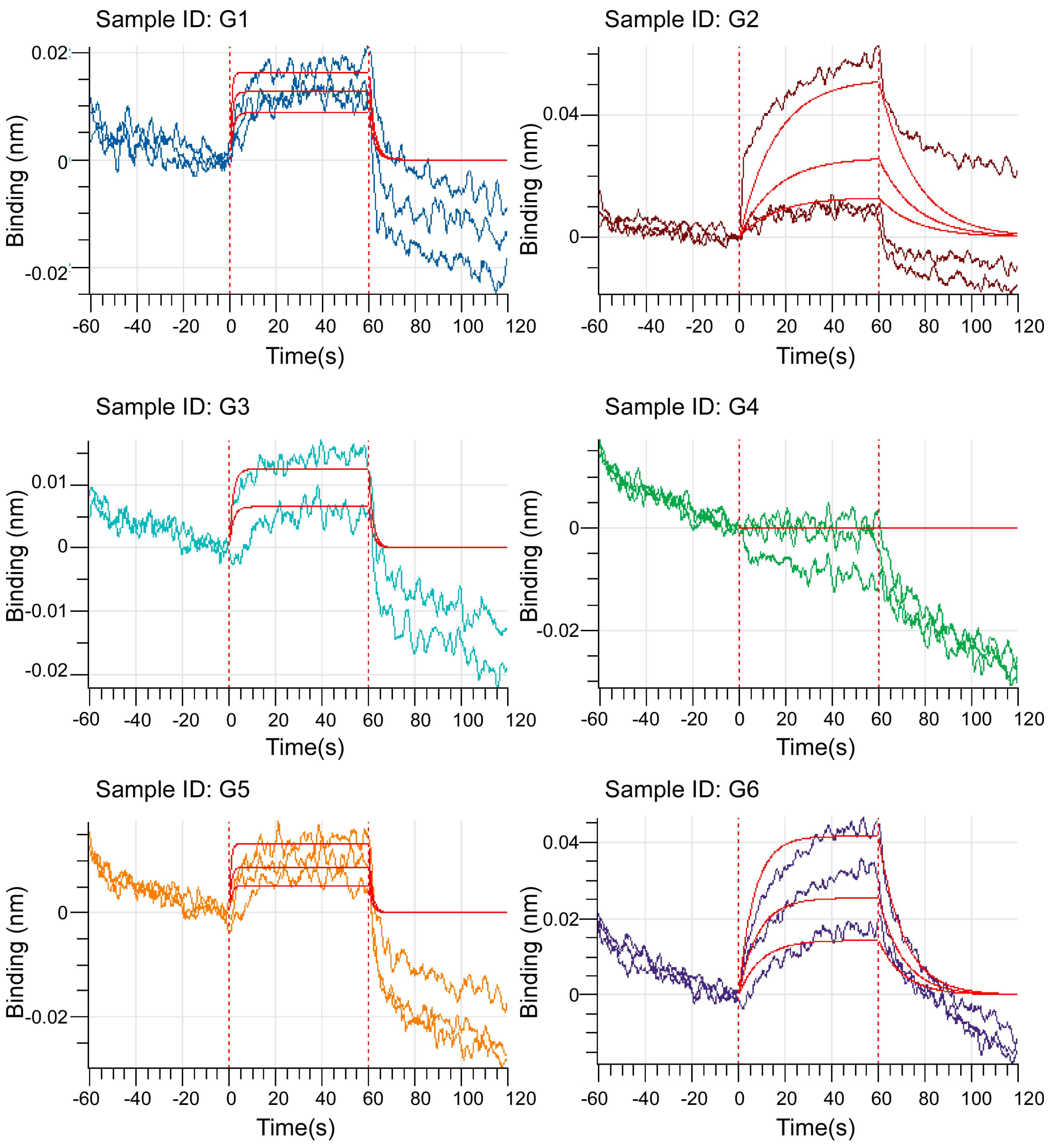

Supplementary Figure S12. Evaluation of the binding capacity of MoApx1 with different MOS. Biomembrane interferometry was employed to evaluate the binding capacity of MoApx1 with different MOS. The sensorgrams display real-time binding responses, with different concentrations of MOS injected over immobilized MoApx1. The resulting curves indicate the binding kinetics, allowing for the calculation of association and dissociation rates.

Table S1. Comparison of mycological characteristics among stains.

Strain	Growth on CM (cm)$^{\alpha}$	Conidiation ($x10^4/cm^2$)$^{\beta}$	Germination rate(%)$^{\chi}$	Appressoria formation (%)$^{\delta}$
Guy11	5.01±0.11A	12.9±0.3A	88.8±3.2A	91.3±2.9A
Δ*Moapx1*	5.00±0.13A	12.7±0.5A	89.3±2.4A	91.2±3.2A
Δ*Moapx1*/ *MoAPX1*	5.00±0.11A	12.8±0.4A	89.1±4.0A	92.0±3.4A

α. Diameter of hyphal radii at day 7 after incubation on CM agar plates at room temperature.
β. Number of conidia harvested from SDC plate at day 10 after incubation at room temperature.
χ. Percentage of conidial germination on artificial surface at 24 h post-inoculation.
δ. Percentage of appressoria formation on artificial surface at 24 h post-inoculation.
Mean and standard deviations (+/- SD) was calculated from three replicates, different letters indicate statistically significant differences ($p < 0.01$).

Table S2. Identification of MoApx1 interacting proteins by Y2H.

Gene ID	Product Description
LOC_Os08g44680.1	photosystem I reaction center subunit II
LOC_Os07g25430.1	photosystem I reaction center subunit IV A
LOC_Os06g46436.1	photosystem I iron-sulfur center
LOC_Os01g31690.1	oxygen-evolving enhancer protein 1
LOC_Os10g21266.1	ATP synthase subunit beta
LOC_Os12g10570.1	ATP synthase subunit beta
LOC_Os07g36080.1	oxygen evolving enhancer protein 3
LOC_Os11g47970.1	AAA-type ATPase family protein
LOC_Os02g33450.1	peroxiredoxin, putative, expressed
LOC_Os06g12580.1	pro-resilin precursor
LOC_Os08g44770.1	copper/zinc superoxide dismutase
LOC_Os08g03520.1	retrotransposon protein, putative, Ty1-copia subclass
LOC_Os01g45274.1	carbonic anhydrase, chloroplast precursor
LOC_Os01g07780.1	embryo-specific 3,putative, expressed
LOC_Os03g17070.1	ATP synthase B chain
LOC_Os04g16740.1	ATP synthase subunit alpha
LOC_Os03g39610.1	chlorophyll A-B binding protein
LOC_Os06g01210.1	plastocyanin, chloroplast precursor

Table S3. Oligonucleotide primers used in this study.

**Primers used for genes deletion**	
pCX62-*MoAPX1*-F1	5'-GGGGTACCGCCATGACGACAGTCGACCG-3'
pCX62- *MoAPX1*-R1	5'-GGAATTCGTTGGATGTGTGTGAAAGAT-3'
pCX62- *MoAPX1*-F2	5'-CGGGATCCATGTTGCACACGTTCTTCTG-3'
pCX62- *MoAPX1*-R2	5'-ATAAGAATGCGGCCGCCGCAGCCGTATCTGCTAGG-3
MoAPX1-KO-F	5'-CGAGGTCGCCAGGTCTGAGAACG-3
MoAPX1-KO-R	5'-TGTACGGCTTGACGTCGTAG-3
MoAPX1-BW-F	5'-TGCCATGACGACAGTCGACCG-3
**Primers used for constriction of complement vectors**	
pYF11-*MoAPX1*–GFP-F	5'-ACTCACTATAGGGCGAATTGGGTACTCAAATTGGTTATGAGGACGACGA CAATAG-3'
pYF11-*MoAPX1*-GFP-R	5'-CACCACCCCGGTGAACAGCTCCTCGCCCTTGCTCACGTTTGGACAGCTA TTTG-3'
pYF11-*MoAPX1*^{L3}–GFP-F	5'-TTGCTTGCCGGGAACGGCCGCATTGTGGCCGCAAGGGACG-3'
pYF11-*MoAPX1*^{L3}-GFP-R	5'-CGTCCCTTGCGGCCACAATGCGGCCGTTCCCGGCAAGCAA-3'
pYF11-*MoAPX1*^{L5}–GFP-F	5'-CGGCGTTCTCAGACCTGGCGGCCTCGTAAAAGATGCTGGC-3'
pYF11-*MoAPX1*^{L5}-GFP-R	5'-GCCAGCATCTTTTACGAGGCCGCCAGGTCTGAGAACGCCG-3'
pYF11-*MoAPX1*$^{\alpha5}$–GFP-F	5'-GTGGAAGGCGGCAAGGGCGGCAAACGTCGAGGCGAAAGCC-3'
pYF11-*MoAPX1*$^{\alpha5}$-GFP-R	5'-GGCTTTCGCCTCGACGTTTGCCGCCCTTGCCGCCTTCCAC-3'
pYF11-*MoAPX1*^{L10}–GFP-F	5'-TGGCGGGTGCGTGCGGGGCAAGGGCGACTCCCGCGACGAG-3'
pYF11-*MoAPX1*^{L10}-GFP-R	5'-CTCGTCGCGGGAGTCGCCCTTGCCCCGCACGCACCCGCCA-3'
pYF11-*MoAPX1*^{L12}–GFP-F	5'-CCGAGTTGGTCGTCTGGGCGGTGCCAAAGACGAGCGGGTT-3'
pYF11-*MoAPX1*^{L12}-GFP-R	5'-AACCCGCTCGTCTTTGGCACCGCCCAGACGACCAACTCGG-3'
pYF11-*MoAPX1*^{L27}–GFP-F	5'-CGATCTTGGGGATCGCCACGGCCTGCGTGTGCACCTTGTG-3'
pYF11-*MoAPX1*^{L27}-GFP-R	5'-CACAAGGTGCACACGCAGGCCGTGGCGATCCCCAAGATCG-3'
pYF11-*MoAPX1*^{L30}–GFP-F	5'-AAGGTGGTGGACCAACCGGCGGCCTCCAAGGCGTCCTTGG-3'
pYF11-*MoAPX1*^{L30}-GFP-R	5'-CCAAGGACGCCTTGGAGGCCGCCGGTTGGTCCACCACCTT-3'
pYF11-*MoAPX1* ^{SBD}-GFP-R	5'-CACCACCCCGGTGAACAGCTCCTCGCCCTTGCTCACGTACGGCTTGAC GTCG-3'
**Primers used for the protein expression *in vivo***	
pBIN-*MoAPX1:GFP*-F	5'-ACGATAGCCGGTACCCCCGGGTGGCCCCAGGA CTCGGAC-3'
pBIN-*MoAPX1:GFP*-R	5'-GCCCTTGCTCACCATCCCGGGGTTTGGACAGCTATT

	TGACAGAGAA-3’
pBIN-*OsPSAD:RFP*-F1	5’-GCAAGAACGTCTTCGACATCATGGACAACACCGAGGACGT-3’
pBIN-*OsPSAD:RFP*-R1	5’-ACGTCCTCGGTGTTGTCCATGATGTCGAAGACGTTCTTGC-3’
pBIN-*OsPSAD*-ORF-F2	5’-GCAAGAACGTCTTCGACATCATGGACAACACCGAGGACGT-3’
pBIN-*OsPSAD*-ORF-R2	5’-GCCCTTGCTCACCATCCCGGGCTACTGGGAGCCGGAGTGG-3’
pBIN-*OsPSAC:HA*-F	5’-GCAGCGGCCGAATTCCCCGGGATGTCACATTCCGTAAAAATTTATGAT-3’
pBIN-*OsPSAC:HA*-R	5’-TCCAAGCTTCTCGAGCCCGGGTCAATAAGATAGAGCCATGCTGCG-3’
pICH86988-*MoAPX1:NYFP*-F1	5’-TTACAATTATCGATACAATGGCAGACCCCGTGTGGCCCCAG-3’
MoAPX1:NYFP-R1	5’-CTCATTAAAGCAGGACCATGATATAGACGTTGTGG-3’
MoAPX1:NYFP-F2	5’-TTACAATTATCGATACAATGATGGCCATGGCCACGCAAGC-3’
pICH86988-*MoAPX1:NYFP*-R2	5’-TCTTCTGCTTGTCGGCCATGATGTCGAAGACGTTCTTGC-3’
pICH86988-*OsPSAD:CYFP*-F1	5’-GCAAGAACGTCTTCGACATCATGGCCGACAAGCAGAAGA-3’
OsPSAD:CYFP-R1	5’-CTCATTAAAGCAGGACTTACTTGTACAGCTCGTCCA-3’
OsPSAD:CYFP-F2	5’-GCAAGAACGTCTTCGACATCATGGCCGACAAGCAGAAGA-3’
pICH86988-*OsPSAD:CYFP*-R2	5’-CTCATTAAAGCAGGACTTACTTGTACAGCTCGTCCA-3’
**Primers used for the protein expression *in vitro***	
pEGX-4T-2- *MoAPX1*-F	5’-CCGGAATTCCCTGGCCCCAGGACTCGGAC-3’
pGEX-4T-2- *MoAPX1*-R	5’-ACGCGTCGACGTTTGGACAGCTATTTGACAG-3’
pEGX-4T-2- MoApx1^{Q26R}-F	5’-TGGCCCAGGGACTCGG-3’
pEGX-4T-2- MoApx1^{Q26R}-R	5’-CCGAGTCCCTGGGCCA-3’
pEGX-4T-2- MoApx1^{A121V}-F	5’-CCACGTCAGCGCTGCGGATCTC-3’
pEGX-4T-2- MoApx1^{L121V}-R	5’-GAGATCCGCAGCGCTGACGTGG-3’
pEGX-4T-2- MoApx1^{L246P}-F	5’-GAACCCGCCCGTCTTTGGCACCAACC-3’
pEGX-4T-2- MoApx1^{L246P}-R	5’-GGTTGGTGCCAAAGACGGGCGGGTTC-3’
pEGX-4T-2- MoApx1^{A394S}-F	5’-CACCGGCTTCTGCCATGGAAGGC-3’

pEGX-4T-2- MoApx1^{A394S}-R	5’-GCCTTCCATGGCAGAAGCCGGTG-3’
pEGX-4T-2- MoApx1^{E397Q}-F	5’-GCCATGCAGGGCTTCACCATC-3’
pEGX-4T-2- MoApx1^{E397Q}-R	5’-GATGGTGAAGCCCTGCATGGC-3’
pEGX-4T-2- MoApx1^{A407T}-F	5’-CCACCACCACGGGACAGGTTCAGAAG-3’
pEGX-4T-2- MoApx1^{A407T}-R	5’-CTTCTGAACCTGTCCCGTGGTGGTGG-3’
pEGX-4T-2- MoApx1^{P466L}-F	5’-CGGTGCTGGTCCTCGAGG-3’
pEGX-4T-2- MoApx1^{P466L}-R	5’-CCTCGAGGACCAGCACCG-3’
**Primers used for qRT-PCR**	
Q-*MoAPX1*-F	5’-CAACAACAACTTCCCCGTGA-3’
Q-*MoAPX1*-R	5’-GAGAGGTCAAAGGTGGTGGA-3’
Q-*OsAOS2*-F	5’-CAATACGTGTACTGGTCGAATGG-3’
Q-*OsAOS2*-R	5’-AAGGTGTCGTACCGGAGGAA-3’
Q-*OsCHT1*-F	5’-CGTGGTGACCAACATCATCA-3’
Q-*OsCHT1*-R	5’-GAGTTGAAAGGCCTCTGGTTGT-3’
Q-*OsACTIN*-F	5’-GCGTGGACAAAGTTTTCAACCG-3’
Q-*OsACTIN*-R	5’-TCTGGTACCCTCATCAGGCATC-3’
Q-*OsPR1a*-F	5’-TCTTCATCACCTGCAACTACTC-3’
Q-*OsPR1a*-R	5’-ATTCATCGGATTTATTCTCACC-3’
**Primers used for Y2H**	
pGADT7-MoApx1$^{\Delta SP}$ –F	5’-GTACCAGATTACGCTCATATGATGAGGACGACGACAATAGC-3’
pGADT7-MoApx1$^{\Delta SP}$ –R	5’-ATGCCCACCCGGGTGGAATTCCTAGTTTGGACAGCTATTTG-3’
pGBKT7-OsPsaD–F	5’-TCAGAGGAGGACCTGCATATGATGGCCATGGCCACGCAA-3’
pGBKT7-OsPsaD–R	5’-TCGACGGATCCCCGGGAATTCCTAGATGTCGAAGACGTTCTTGCC-3’
pGBKT7-OsPsaC–F	5’-TCAGAGGAGGACCTGCATATGATGTCACATTCCGTAAAAATTTATGAT-3’
pGBKT7-OsPsaC–R	5’-TCGACGGATCCCCGGGAATTCTCAATAAGATAGAGCCATGCTGCG-3’
