# Supplementary figures and images for: The multifunctional ascorbate peroxidase MoApx1 secreted by Magnaporthe oryzae mediates the suppression of rice immunity
Source: Plant Cell. 2025 Jun 11;37(7):koaf146. doi: 10.1093/plcell/koaf146 (PMC12231552; doi:10.1093/plcell/koaf146)

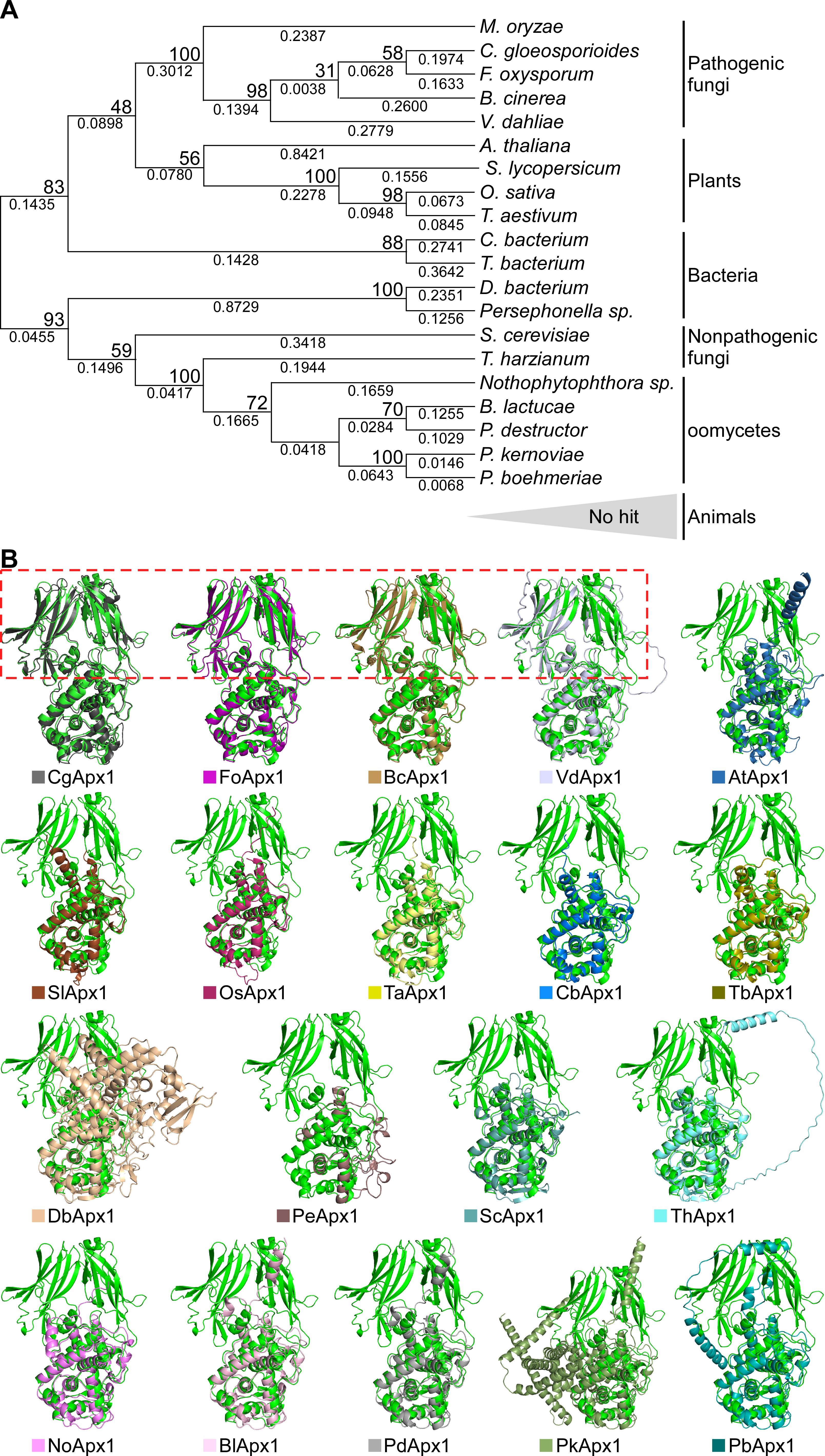

Supplement: koaf146_Supplementary_Data [file koaf146_supplementary_data.zip › Figure S10.tif]

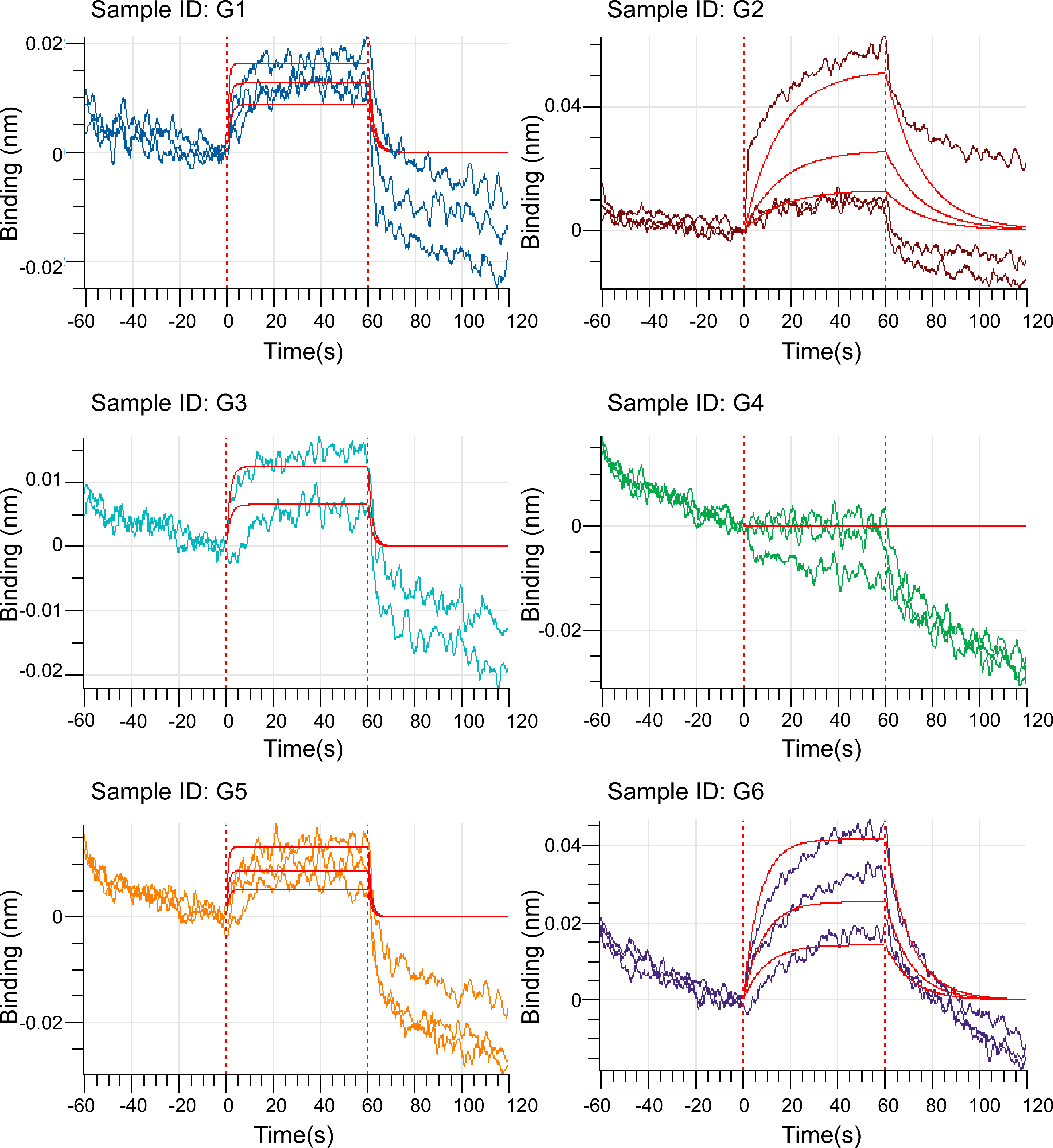

Supplement: koaf146_Supplementary_Data [file koaf146_supplementary_data.zip › Figure S12.tif]

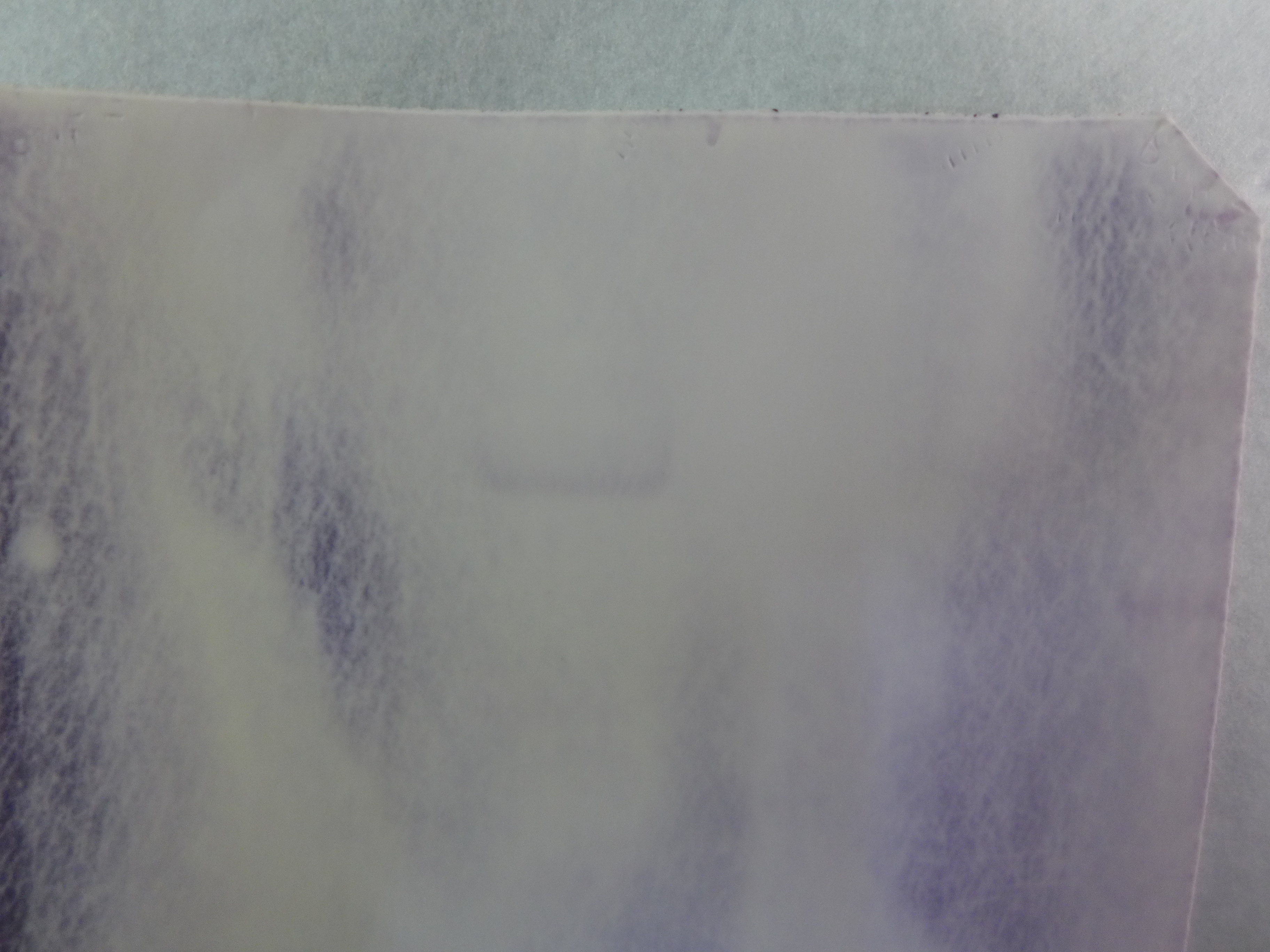

Supplement: koaf146_Supplementary_Data [file koaf146_supplementary_data.zip › Figure S2B left lane.JPG]

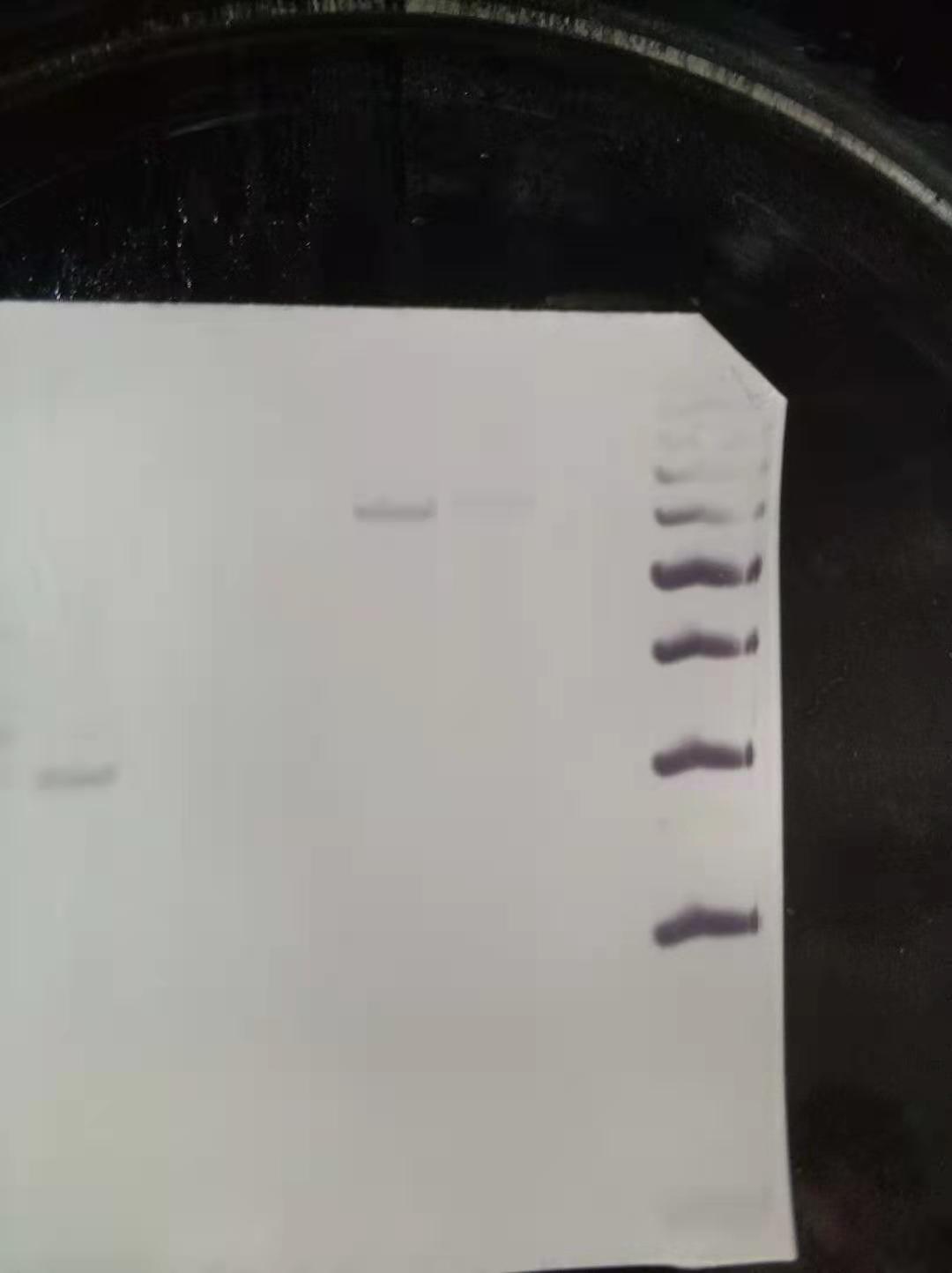

Supplement: koaf146_Supplementary_Data [file koaf146_supplementary_data.zip › Figure S2B right lane.jpg]

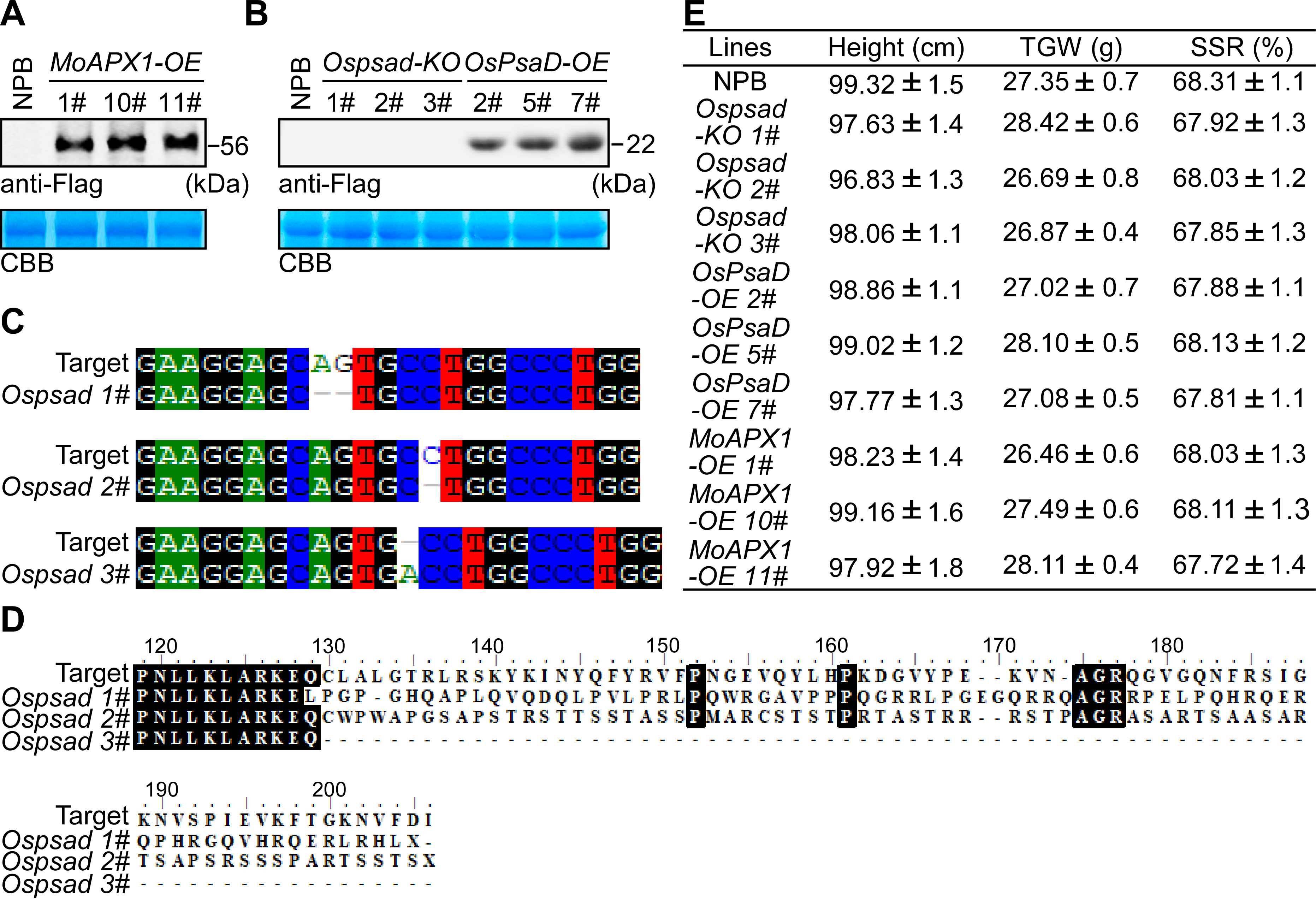

Supplement: koaf146_Supplementary_Data [file koaf146_supplementary_data.zip › Figure S4.tif]

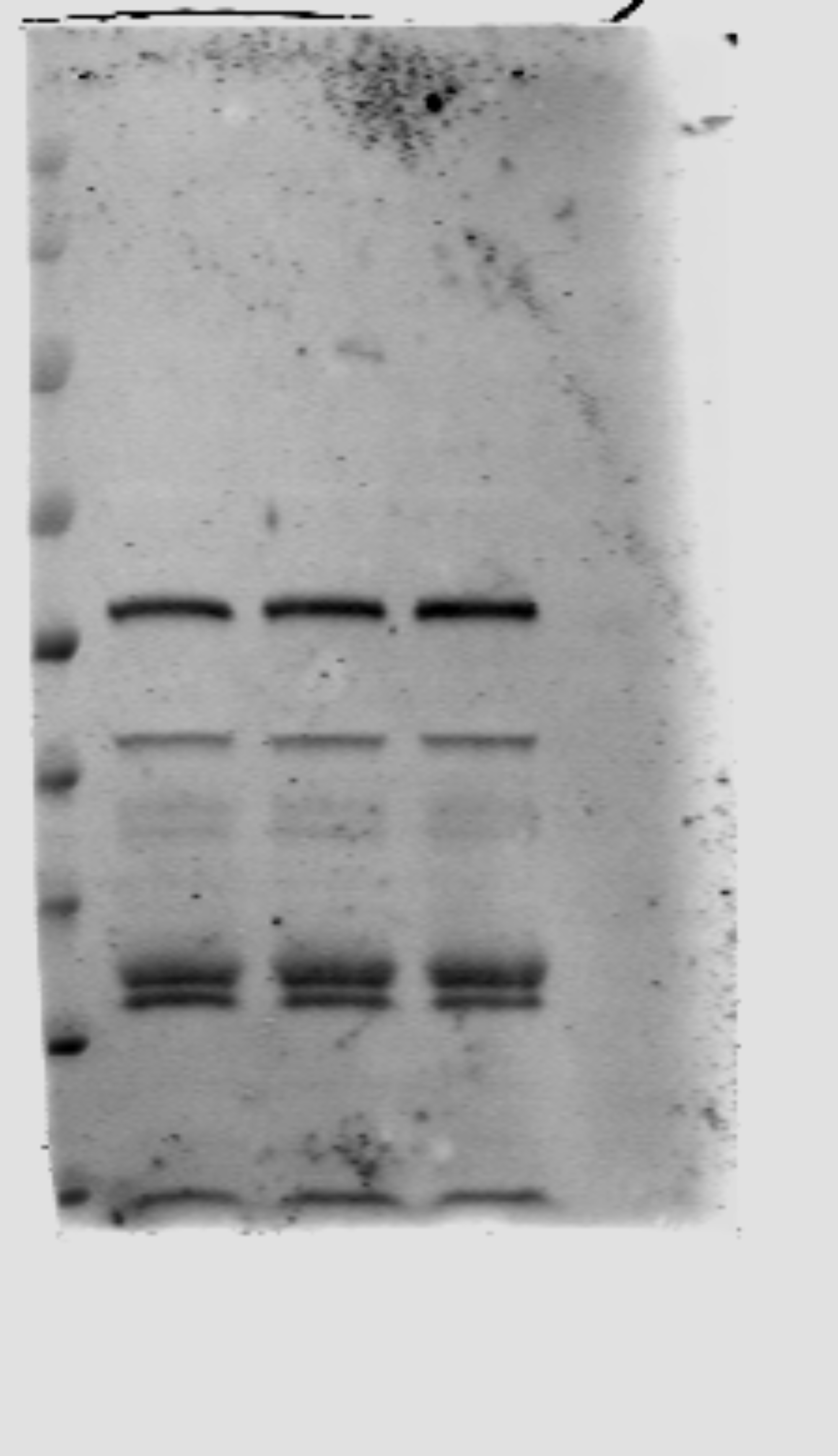

Supplement: koaf146_Supplementary_Data [file koaf146_supplementary_data.zip › Figure S4A.tif]

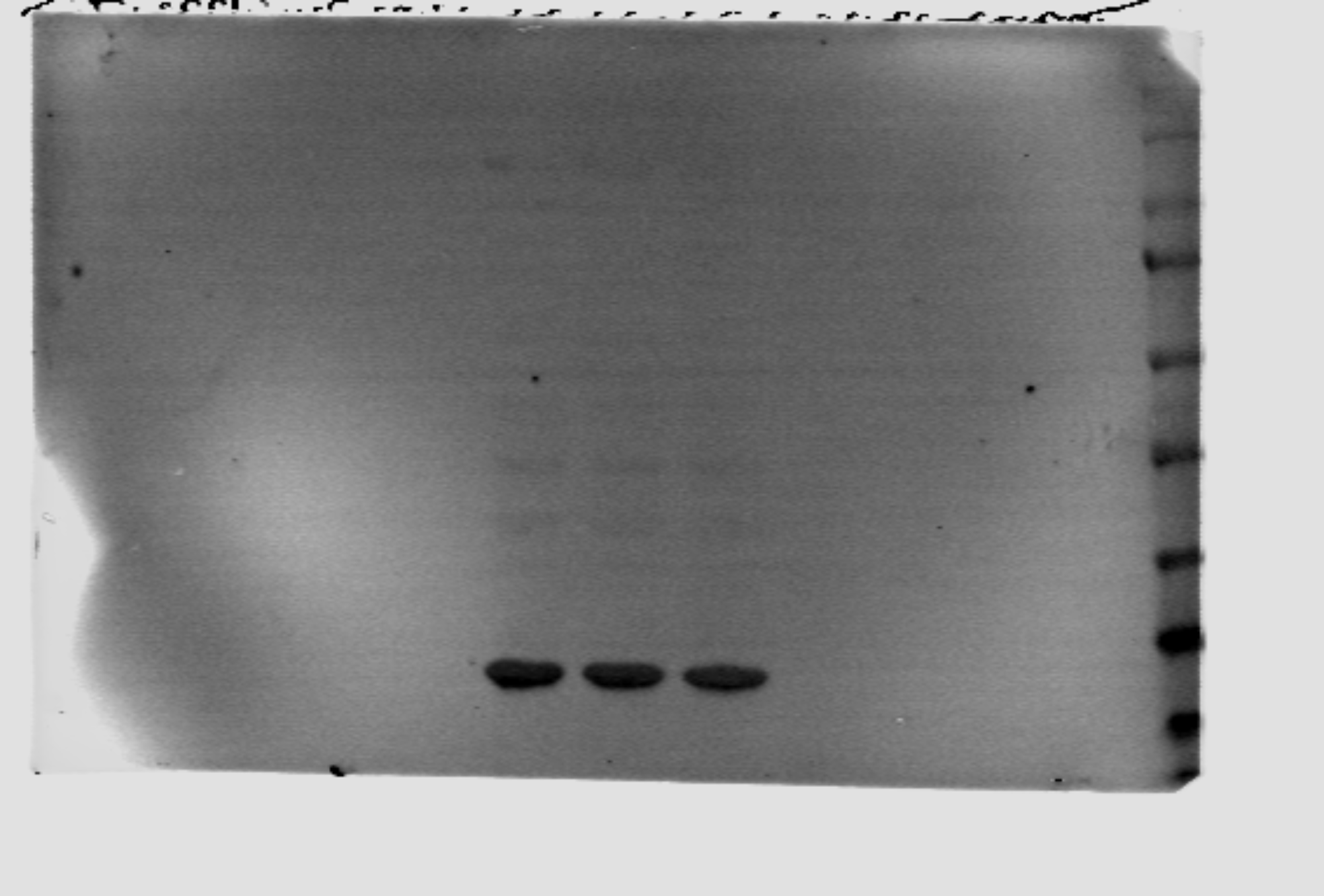

Supplement: koaf146_Supplementary_Data [file koaf146_supplementary_data.zip › Figure S4B.tif]

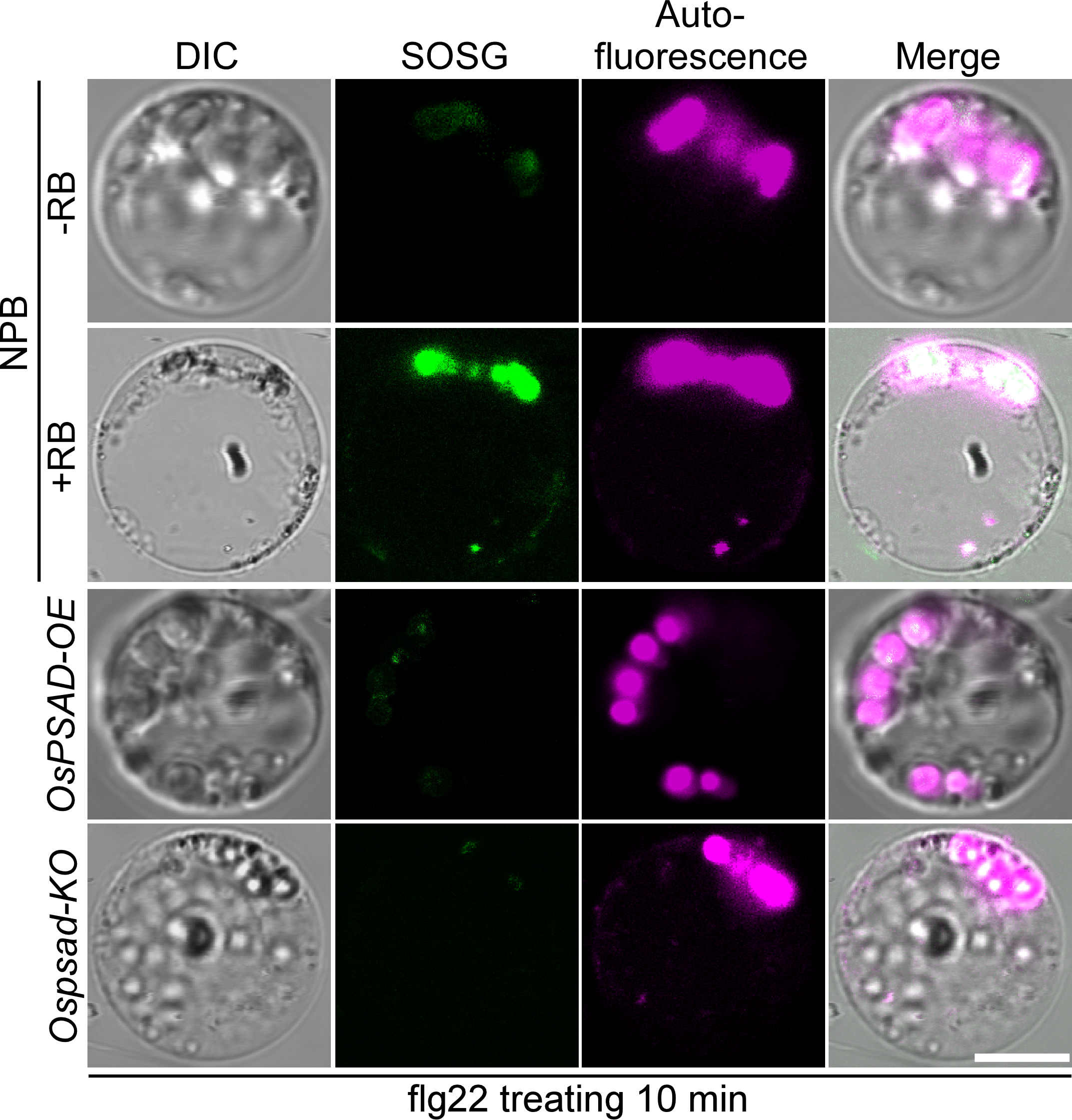

Supplement: koaf146_Supplementary_Data [file koaf146_supplementary_data.zip › Figure S8.tif]
